# Human Amniotic Epithelial Cells Secretome: Components, Bioactivity, and Challenges

**DOI:** 10.3389/fmed.2021.763141

**Published:** 2022-01-10

**Authors:** Ibrahim Fathi, Toshio Miki

**Affiliations:** Department of Physiology, Nihon University School of Medicine, Tokyo, Japan

**Keywords:** human amniotic epithelial cells (hAECs), secretome, exosomes, conditioned media (CM), paracrine, cell-free therapeutics

## Abstract

Human amniotic epithelial cells (hAECs) derived from placental tissue have received significant attention as a promising tool in regenerative medicine. Several studies demonstrated their anti-inflammatory, anti-fibrotic, and tissue repair potentials. These effects were further shown to be retained in the conditioned medium of hAECs, suggesting their paracrine nature. The concept of utilizing the hAEC-secretome has thus evolved as a therapeutic cell-free option. In this article, we review the different components and constituents of hAEC-secretome and their influence as demonstrated through experimental studies in the current literature. Studies examining the effects of conditioned medium, exosomes, and micro-RNA (miRNA) derived from hAECs are included in this review. The challenges facing the application of this cell-free approach will also be discussed based on the current evidence.

## Introduction

Perinatal stem cells are a group of cells that are derived from the extra-embryonic tissues, including the fetal membrane, amniotic fluid, and umbilical cord. This unique group of cells combines the characteristics of adult stem cells, e.g., mesenchymal stem cells, and the differentiation potential of embryonic stem cells (1). In addition, these cells are considered immune-privileged, causing no adverse inflammatory reactions in recipient animal models. Their genetic stability, as compared to embryonic stem cells, is evidenced by the absence of teratoma formation in animal models (2).

These attractive features, in addition to the ability of isolation from tissues that are normally discarded, made perinatal stem cells a safe and practical option for clinical cell transplantation approaches and obviated ethical debates (2, 3). Various cell types were isolated from perinatal tissues. Both mesenchymal and epithelial cells were isolated from the amniotic component of the fetal membrane (i.e., amniotic membrane) and were investigated for their therapeutic potential. The epithelial component, however, is derived from pluripotent epiblasts, while the mesenchymal component belongs to hypoblast-derived mesoderm. In addition, the epithelial component is more suitable for clinical application because of its compliant isolation protocol and large cell yield (2, 3).

**Figure 1:**
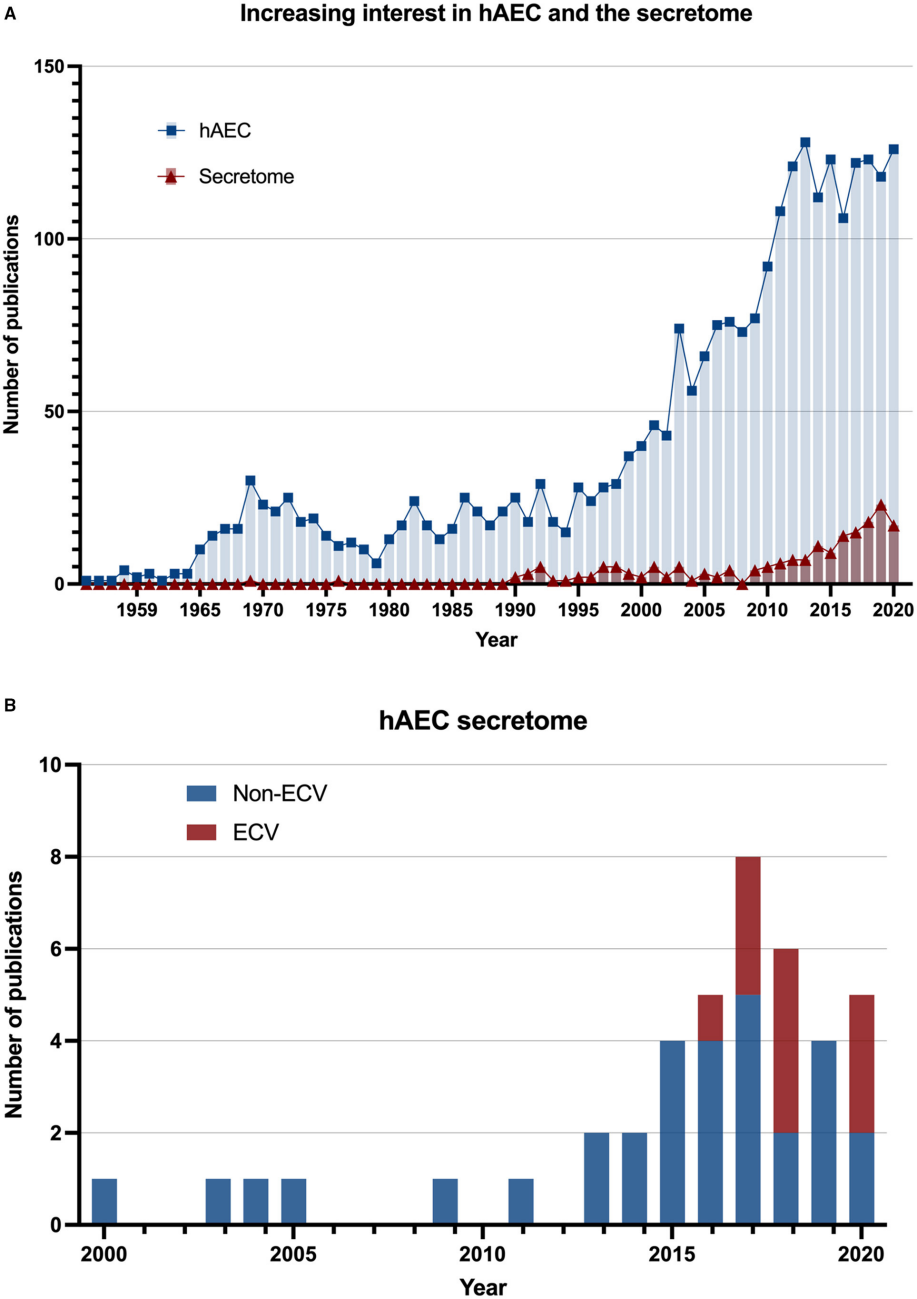
Increasing interest in amnion epithelial cells secretome. **(A)** The number of scientific publications by year from 1941 to 2020. Data were searched in the PubMed database using the keywords “human amniotic epithelial cells” (square: blue) and “human amniotic epithelial cells AND (paracrine) OR (extracellular vesicles) OR (exosomes) OR (conditioned medium)” (triangle: red). **(B)** The number of scientific publications that have studied human amniotic epithelial cell secretome since 2000. The stacked bar chart indicates inclusion of extracellular vesicles (ECVs) in the secretome studies.

As a result, the human amniotic epithelial cells (hAECs) were the focus of extensive research during the last two decades. The therapeutic potential of transplanted hAECs was examined in different pathological conditions in animal models including liver, epidermal, cardiac, ophthalmological, ovarian, musculoskeletal, and neurological conditions, and was shown to improve wound healing and tissue repair, together with having anti-fibrotic and anti-inflammatory effects. These studies of hAEC transplantation were comprehensively reviewed elsewhere (4). Several studies indicated that a large proportion of these therapeutic effects was retained in the hAEC-derived conditioned medium (CM) or hAEC-derived extracellular vesicles (ECVs), and were therefore considered to be of paracrine nature (5–7). Thus, both CM and ECV preparations have strongly emerged as potential cell-free therapeutic tools (Figure 1).

In this article, we describe the different components of hAEC-secretome and comprehensively review the studies that examined its therapeutic potential in different animal models and discuss the significant findings in the *in vitro* studies. We also highlight the hurdles facing the progress of this cell-free approach and the possible methods for clinical application.

## Human Amniotic Epithelial Cell Secretome

The secretome can be defined as the “entirety of soluble paracrine factors released by cells in the conditioned medium” (8). Although this definition excludes the ECVs, their influence cannot be excluded in the studies utilizing CM unless ECVs were entirely removed, or their production was specifically inhibited. One can also argue that ECVs, which are secreted by cells into the extracellular space (9), should be included in the term “secretome.” Therefore, in this article, we describe the hAEC-secretome including its hAEC-ECV component.

Extracellular vesicles (ECVs) are extracellular microvesicles that can be classified into exosomes, ectosomes (microvesicles or microparticles), and apoptotic bodies (10). Exosomes are released from cells upon fusion of an intermediate endocytic compartment, the multivesicular bodies, with the plasma membrane, liberating the intraluminal vesicles into the extracellular space, whereas ectosomes bud out directly from the plasma membrane (10). On the other hand, the apoptotic bodies are derived from cells undergoing apoptosis (10). The three types fall within the sub-micrometer size range and are impossible to completely isolate one purified category using the classical differential centrifugation technique. As a result, this differentiation is commonly overlooked, and the studies are conducted using the mixed ECV compartment. In a position statement released from the International Society for Extracellular Vesicles (ISEV) in “Minimal information for studies of extracellular vesicles (MISEV 2018)” (11), the society recommends using the term “extracellular vesicles” unless specific markers for ECV subcategories could be illustrated. It is also important to mention that the ECVs vary in yield and, more importantly, purity depending on the method of isolation (12). As expected, the proteomic profile was also reported to vary according to the isolation method (13). The different methods of ECV isolation and their impact on ECV yield and purity are described elsewhere (14).

## Profiling of hAEC-Secretome

Several investigators attempted to dissect the content of hAEC-secretome either through target cytokine analysis or comprehensive proteomic and micro-RNA (miRNA) profiling.

### Extracellular Vesicles Profiling

**Figure 2:**
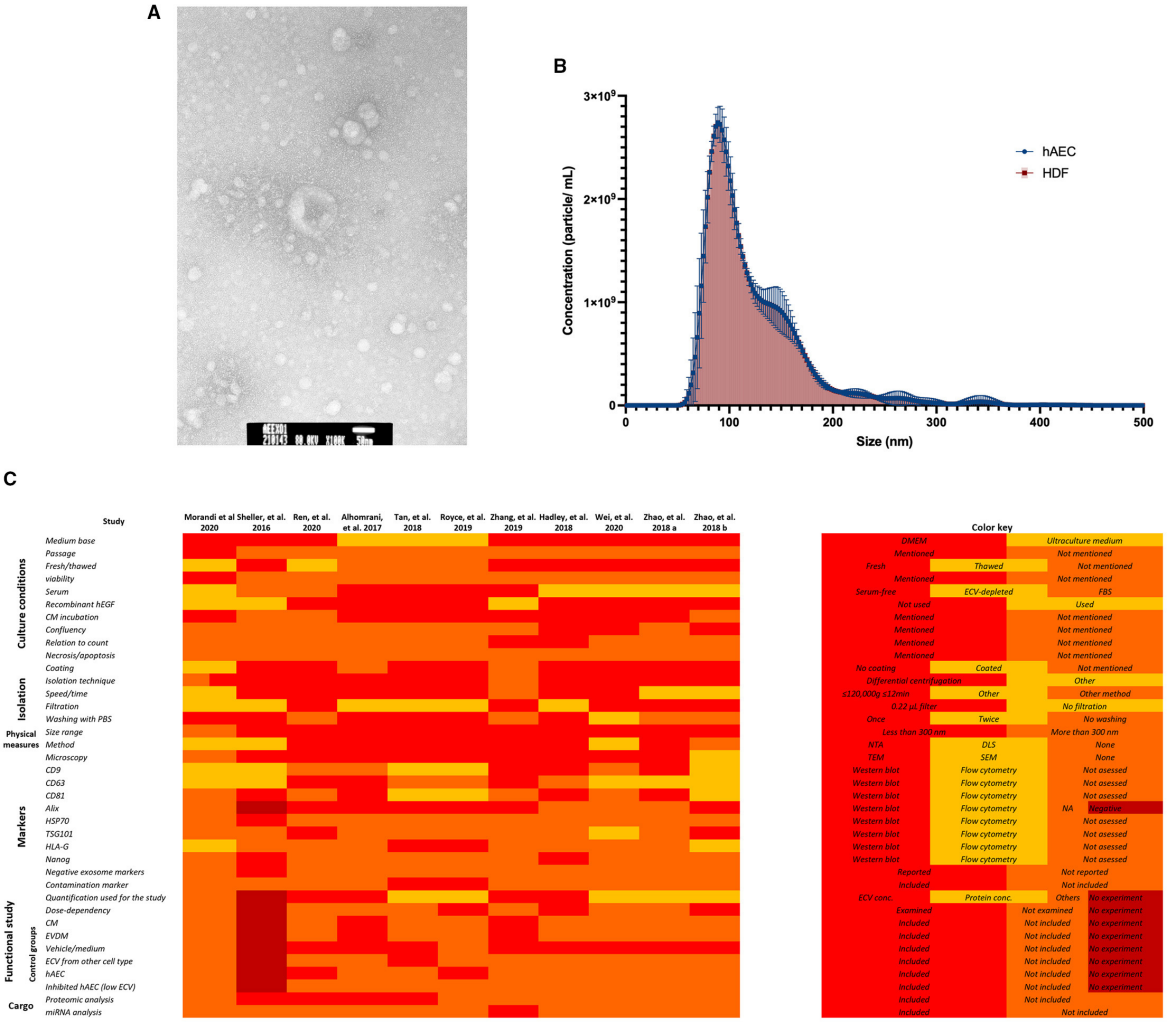
Example of amnion epithelial cell–derived ECV characterization. **(A)** Representative transmission electron microscopy image. hAEC-ECVs reveal cup-shaped morphology. Scale bar: 50 nm. **(B)** Size distribution of human amniotic epithelial cell-extracellular vesicles (hAEC-ECVs) obtained by Nanoparticle tracking analysis. The number of particles of hAEC-derived and human dermal fibroblast (HDF)-derived exosomes were plotted as mean ± SD (circle, blue) and mean ± SD with droplines (square, red), respectively. **(C)** Variation in methodologies of hAEC-ECV isolation and characterization.

Human amniotic epithelial cells-extracellular vesicles (hAEC-ECVs) were most commonly isolated using differential centrifugation, with or without filtration through a 0.22-μm filter. The protocols to produce ECV mainly used serum-free media, although 10% serum or exosome-depleted serum were also used in some studies. The peak size of ECVs, as analyzed by nanoparticle tracking, most commonly fell in the range of 100–150 nm. Morphological assessment by transmission electron microscopy demonstrated a typical cup-shaped morphology in most of the studies. Using a combination of Western blotting or bead-based flow cytometry were used to demonstrate surface or internal markers of ECVs (e.g., Alix, CD81, CD9, and HLA-G). Although the majority of the studies in this review have adhered to the MISEV characterization and reporting criteria, the validation of ECV purity through the detection of non-ECV markers was commonly neglected, which can influence the results of the functional experiments (11). On the contrary, electron microscopy was commonly used for characterization, despite that nanoparticle tracking analysis provides the biophysical information of ECV. Figure 2 shows an example of ECV characterization and demonstrates the variation in isolation and characterization methodologies.

The content, or cargo, of ECVs includes lipids, nucleic acids, and proteins (9). Several studies reported the protein and miRNA profile of hAEC-ECVs. Comprehensive proteomic analysis of hAEC-ECVs identified 171 proteins in one study (15). Functional annotation according to DAVID functional enrichment analysis tool yielded annotation of a large portion into either extracellular or membrane origin, with a large contribution to the cytosol and endoplasmic reticulum (ER) lumen. These proteins were mainly associated with protein, heparin, and cadherin binding, and extracellular matrix structural constituents. The most enriched biological processes included cell adhesion, leukocyte migration, angiogenesis, innate immune response, regulation of complement activation, positive regulation of cell proliferation, and extracellular matrix organization.

Comparative analysis using liquid chromatography (LC) followed by mass spectrometry (MS) showed that 84 proteins that can be associated with the Reactome pathway were significantly different in the hAEC-ECVs as compared to human lung fibroblast-derived ECVs (16), and 164 proteins were unique to hAEC-ECVs in comparison to CM and ECV-depleted CM (EVDM), while 51 components were in common in another study (17).

Gene ontology (GO) analysis of the hAEC-ECV protein cargo showed enrichment of biological processes associated with protein transport, cell-cell adhesion, receptor-mediated endocytosis, cell surface receptor signaling pathway, integrin-mediated signaling pathway, wound healing, and membrane organization (17). Reactome pathway analysis of hAEC-ECV proteins was enriched in pathways involved with apoptosis, MAP kinase, developmental growth, inflammation-mediated pathway, EGF, PDGF, and FGF signaling in one study (16), while the Kyoto Encyclopedia of Genes and Genomes (KEGG) pathway analysis indicated the hAEC-ECV enriched proteins associated with Rap1 signaling, extracellular matrix (ECM)-receptor interaction, focal adhesion, antigen processing and presentation, and PI3K-Akt signaling pathway (17). Examples of the unique hAEC-ECV proteins in comparison to EVDM and the significantly enriched Reactome pathways in hAEC-ECVs in comparison to ECVs from human lung fibroblasts are shown in Table 1. Typical ECV markers were also found in abundance including tetraspanins CD9 and CD81, various Rab GTPases, and select components associated with vesicle sorting/trafficking including ARF1, LAMP1, and CLTC (17). hAEC-ECVs contained 61 proteins that were identified in the exosome database ExoCarta in the top 100 highly expressed proteins in exosomes (17). In addition to variations in proteomic analysis pipelines and bioinformatics approach, analysis variations can also be ascribed to the different contaminants of ECVs from culture media. Furthermore, a recent study demonstrated a variation in results of the proteomic analysis in response to different hAEC culture media formulations (18).

**Table 1:** Proteomic analysis of human amniotic epithelial cells-extracellular vesicles (hAEC-ECVs).

**(A) Unique proteins in ECV vs. EVDM**	**GO category**	**(B) Top represented KEGG pathways**	**Number of genes**	**(C) Significantly overrepresented Reactome pathways**
Programmed cell death protein 6 (Apoptosis-linked gene 2 protein homolog) (ALG-2)	Sorting/Trafficking	hsa04151:PI3K-Akt signaling pathway	19	Cellular response to heat stress
Histone H2A	Cell adhesion			
CD81 tetraspanin	Tetraspanin	hsa04510:Focal adhesion	16	HSF1-dependent transactivation
Complement decay-accelerating factor (fragment)	Complement factor			
Integrin beta	Integrin	hsa04015:Rap1 signaling pathway	11	Attenuation phase
CD59 glycoprotein	Complement factor, GPI anchor			
Prostate stem cell antigen	GPI anchor	hsa04512:ECM-receptor interaction	8	Regulation of HSF1-mediated heat shock response
Protein S100-A6 (Calcyclin) (Growth factor-inducible protein 2A9) (MLN 4) (Prolactin receptor-associated protein) (PRA) (S100 calcium-binding protein A6)	S100 proteins			
Integrin beta-4 (GP150) (CD antigen CD104)	Wound response, integrin	hsa04612:Antigen processing and presentation	7	HSF1 activation
Ras-related C3 botulinum toxin substrate 1 (Cell migration-inducing gene 5 protein) (Ras-like protein TC25) (p21-Rac1)	Wound response			

***(A)** Examples of proteins unique to hAEC-ECVs in comparison to ECV-depleted medium (EVDM), **(B)** top 5 represented KEGG pathways of hAEC-ECV proteins, **(C)** significantly overrepresented Reactome pathways in hAEC-ECVs as compared to human lung fibroblast ECVs. Source: **(A,B)** (17); supporting information, and **(C)** (16); supporting information*.

**Figure 3:**
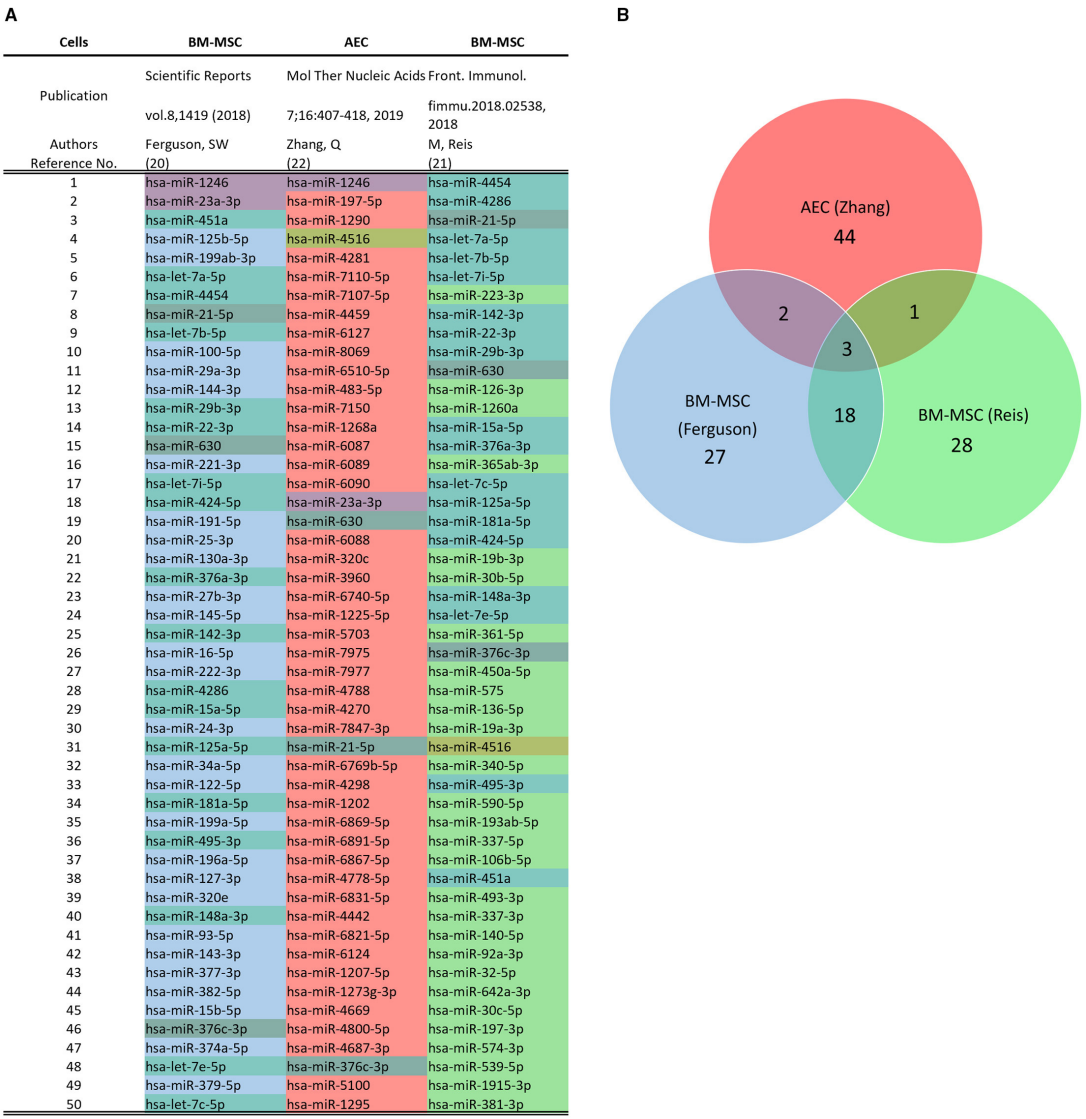
Comparison of microRNA Profile of hAEC-ECVs and mesenchymal stem cell (MSC)-ECVs. **(A)** Top 50 miRNA in hAECs and MSC-ECVs as detected using the NanoString platform in the respective studies (20–22). **(B)** Overlap between miRNA content from hAECs and MSC-ECVs.

Regarding the miRNA cargo, RNA-Seq followed by Priori analysis of gene union enrichment, followed by ranking with respect to the number of genes targeted in the significantly overrepresented pathways, showed enrichment in signaling pathways associated with fibrosis, including PI3K-Akt, MAPK, Ras, TGFb, Hippo, and focal adhesion signaling pathways. Predicted targets of the miRNA cargo identified through a posteriori analysis included 110 genes associated with the KEGG pathway “proteoglycans in cancer,” followed by genes associated with Hippo signaling and stem cell pluripotency pathways. Among the identified miRNA in ECV cargo, known anti-fibrotic miRNAs were demonstrated, including miR-23a, miR-203a, miR-150, and miR-194 (16). In another study, micro-RNA array and bioinformatics were used to identify the hAEC-ECV miRNA cargo (19). The statistically over-represented pathways and biological processes were mainly related to the phosphatidylinositol signaling system, proliferator-activated receptor (PPAR) signaling pathway, and apoptotic process involved in morphogenesis. Figure 3 shows a comparison between the top 50 miRNA in hAECs and MSC-ECVs (20–22). qRT-PCR showed that has-miR-1246 was highly expressed in hAEC-ECVs. In another study (23), miRNA cargo of ECVs was profiled and showed the high enrichment of the PI3K-AKT pathway. P-AKT and p-mTOR expression levels were confirmed to be significantly increased in ECV treated cells (hUVECs and human fibroblasts) by Western blotting. hAEC-ECVs obtained under oxidative stress showed higher colocalization of high mobility group box 1 (HMGB1) and cell-free fetal telomere fragments (cffTFs) compared to those obtained using standard culture medium (24). In addition, Next-generation sequencing (NGS) showed that ECV cargo includes genomic and mitochondrial DNA (24).

### Soluble Factors Profiling

The profiling of CM and ECV-depleted CM (EVDM) using GeLC-MS/MS was reported by Alhomrani et al. (17) and was finely presented in the “supporting information.” Two studies used a cytokine array to examine 507 human cytokines to profile the components of hAEC-CM. In comparison to basal medium, 109 cytokines showed more than a 2-fold change in hAEC-CM (6). Analysis showed that most secretory factors had a relatively higher expression in hAEC-CM in comparison to CM from adult foreskin fibroblast culture (hEF-CM) (25). Another array of 40 inflammatory cytokines showed that IL-8, MCP-1, RANTES, MIP-1b, TGF-β1, TNF, and IL-13 had the highest expression levels in hAEC-CM (26), while a 41 growth factor array (27) showed that CSF2, CSF3, HB-EGF, IGFBP-2, CSF1R, PDGF, and TGF-β1 had the highest expression levels in hAEC-CM. When the effect of culturing conditions was examined, ≥1.5-fold increase of ANG, EGF, IL-6, and MCP-1 was noted when hAECs were cultured under hypoxic conditions as compared to normal conditions (27). This finding was confirmed by ELISA and mRNA expression. GO enrichment analysis showed enrichment in the regulation of apoptosis (37 proteins), immune response (34 proteins), angiogenesis (24 proteins), and regulation of cell cycle (16 proteins) in one study (6), while the other study (25) reported enrichment in the biological processes associated with positive regulation of locomotion, cell migration and, chemotaxis, cellular component movement and response to external stimulus, and tyrosine modification activity. Meanwhile, the KEGG pathway enrichment analysis showed enrichment of chemokine, cytokine-cytokine receptor, JAK-STAT, Pi3KAkt, MAPK, Ras, Rap1, and TGF-beta signaling pathways (25).

Specific targets of interest in hAEC-CM were analyzed in several studies. Examples include the high expression of neutrophilic factors, brain-derived neurotrophic factor (BDNF), ciliary neurotrophic factor (CNTF), neuronal cell adhesion molecule (NrCAM), and in hAEC-CM in comparison to hEF-CM (25). BDNF and NT-3, but not NGF and CNTF, were detected by ELISA in another study (28). The concentration of BDNF and NGF were 252.7 and 140.4 pg/ml, respectively (29). In addition, various anticancer-related cytokines were detected in hAEC-medium including TGF-β1, granulocyte/macrophage colony-stimulating factor (GM-CSF), interleukin-8 (IL-8), IL-6, IL-1, interferon-γ (IFN-γ), tumor necrosis factor-α (TNF-α), TGF-β2, and Smad 7 (30).

Other detected cytokines included prostaglandin E2 (PGE2) (43.7 × 10^5^ pg/ml, for comparison, CM from hepatic stellate cells contained an average of 11.9 pg/ml), TGF-β3 (150 pg/ml) (31), interleukin-1 receptor antagonist (IL-1ra), and oncostatin M (OSM) (25). On the other hand, Bone morphogenetic protein-7 (BMP-7), interleukin-10 (IL-10), FAS-ligand, relaxin (31), EGF (28), and bFGF (28, 32) were not detected, while tumor necrosis factor-related apoptosis-inducing ligand (TRAIL) was detected but below the quantifiable range of the assay (31).

## Biological Effects of hAECs Secretome

A comprehensive search through the PubMed database, MEDLINE database, and Google Scholar resulted in 42 original studies that examined the paracrine action of hAECs (summarized in Tables 2, 3). One article was excluded because it was only available in Chinese.

**Table 2:** *In vitro* studies demonstrating the paracrine effect of hAECs.

**Cells**	**Preparation**	**Significant findings**	**References**
HK-2 cells	ECV	- Protection from hypoxia reoxygenation injury-induced apoptosis (Western blot: cleaved Caspase 3). - Improved their proliferation (mRNA: PCNA).	(15)
Mouse BM macrophages	ECV	- Increase in M2 markers (CD206, CD163, IL4rα, and Arg1 mRNA) and downregulation of M1 markers (CD86, IFNγ, TNFα, and iNOS mRNA) after 7 days of culture.	(15)
	ECV, CM, or EVDM	- Increased M2 marker (CD206, FC) in the M1 induction group, while no significant influence was noted in the M2 induction group or naïve macrophages group.	(17)
	CM	- Exposure CM, with or without pre-stimulation with LPS, reduced the proportion of CD86+ cells and increased the proportion of CD206+ cells (Flow cytometry). - qPCR showed a modest increase in M2-specific genes after LPS exposure, which was further increased when CM as compared to LPS-exposed control. - Reduced chemotaxis of macrophages toward rMIP-2 and increased their phagocytic ability. - No significant effect on macrophage proliferation, regardless of exposure to LPS.	(33)
	ECV	- Treatment with 5 μg hAEC-ECV showed double the phagocytic activity in comparison to control (effect of HLF- was remarkably lower than hAEC-ECV but not significant). - Higher percentage of CD206+ (M2 marker) and lower CD86+ (M1 marker) cells in comparison to control and HLF-ECV.	(16)
	CM	- Increased M2 marker (CD206) and decreased M1 marker (iNos) in macrophages treated with TNF-α + INF-γ.	(26)
Human endometrial MSCs damaged by H_2_O_2_	Trans-well hAEC coculture	- Decreased p62 protein level (WB), an indicator of autophagic flux inhibition. - Increased the LC3-II/LC3-I ratio (WB). - Restored the levels of estrogen receptor (ER, WB).	(19)
Myometrial, decidual, and placental cells	ECV from oxidatively stressed hAEC	- ECV from normal or stressed hAEC increased the secretion of IL-6, IL-8, and PGE2 (ELISA) in myometrial and decidual cells. - Increased activation of NF-κβ (WB) in myometrial and decidual cells. - No effect was observed on the level of IL-6, IL-8, PGE2, IL-1β, or TNF-α in placental cells.	(34)
hUVECs, hGL, and hFB	hAEC coculture using Boyden chamber	- Several features of chemotherapy damage of hGL cells were reversed coculture including improved cell count (CCK-8 assay), decreased Annexin-V (+)/PI (+) cells, and decreased cleavage of Caspase 3 expression. - Cocultured hGL showed higher expression of P-SMAD2 and P-SMAD3 proteins (WB) in comparison to chemotherapy-treated group. - Coculture increased the mean tube length of hUVECs.	(6)
	CM	- Increased hUVEC proliferations (CCK8) and migration (trans-well assay).	(26)
	ECV	- Promoted the proliferation of hUVECs and hFB. - Increased the migration of hFB (scratch assay and trans-well assay). - Increased the number of capillary-like structures were formed by hUVECs (tube-formation assay). - LY294002 to selectively block the PI3K-AKT-mTOR pathway significantly weakened the previous effects.	(23)
KGN cells	ECV	- Inhibited chemotherapy-induced granulosa cell apoptosis; shown by decreasing Bax and cleaved Caspase 3 and increased anti-apoptotic genes, Bad and Bcl2, expression (WB). - Similar reversal of chemotherapy-induced cleaved Caspase 3 elevation was seen when granulosa cells were transfected with miR-1246 or miR-21-5p mimic.	(19)
Mouse RAW264.7 macrophages	CM	- CM inhibited the pro-inflammatory markers TNF-α, IL-6, and iNOS-2 and increased expression of IL-10 and CD206 genes after LPS treatment. - In the absence of LPS, no significant effect of CM on these markers was noted. - CM inhibited the migration of RAW264.7 cells that was induced by LPS in a macrophage migration assay.	(7)
HSCs	CM	- Reduction of myofibroblast markers α-SMA (qPCR), TGF-β1 expression (ELISA), and collagen production ([3H] proline incorporation). - 2.74-fold increase in matrix metalloproteinase-9 (MMP-9) gene expression. - CM had a negative influence on HSC proliferation (BrdU and platelet-derived growth factor-β expression) and increased their apoptosis by (11.8%, Annexin V:PI FC).	(31)
LX2	ECV or CM	- Collagen production ([3H] proline incorporation) decreased when treated with ECV or CM.	(17)
LPC	CM	- Increased BrdU incorporation in liver progenitor cell (LPC) cultures after 3 days of treatment. - After 8 days of culture, CM induced differentiation toward hepatocytes as demonstrated by increased AFP and albumin expression. - Activity of tyrosine aminotransferase promoter was only observed in hAEC-CM treated LPCs - Urea production in CM-treated group was higher than untreated and hepatocyte-differentiation-medium-treated cultures after 16 days. - KEGG pathway analysis of microarray showed that genes associated with drug, glutathione, and arachidonic acid metabolism, steroid biosynthesis, and bile acid secretion were upregulated in LPC cultures, while genes associated with cell proliferation and phosphoinositide 3-kinase signaling were downregulated. - Gene Ontology (GO) pathway analysis showed that the upregulated genes were significantly enriched in multiple metabolic processes, negative regulation of cell growth, and apoptosis, while downregulated genes were enriched in pathways related to cell proliferation.	(5)
Human dermal fibroblasts	CM	- α-SMA, Col-I, and Col-III induced by TGF-β1 (qPCR and WB) were significantly repressed in the TGFβ1+ CM co-treatment group compared to TGF-β1 alone. - CM showed similar results in fibroblasts derived from keloid - After TGF-β1 stimulation of human dermal fibroblasts, CM significantly increased MMP-1, and TIMP-1 (qPCR and WB). - CM attenuated the TGF-β1-induced contraction of F-actin bundles and decreased the number of bundles. - CM addition to keloid fibroblasts also resulted in a reduction in supernatant collagen. - MMP-1, MMP-2, and TIMP1 were upregulated in keloid fibroblasts by CM.	(35)
	ECV	- Enhanced the proliferation of fibroblasts. - Remarkably increased their migration in a scratch assay in a dose-dependent manner. - Col-I and Col-III were repressed by 100 μg/mL ECV. - Collagen in the supernatant (Sircol collagen assay) was reduced by 100 μg/mL ECV. - MMP-1 mRNA was upregulated	(36)
	ECV ± PROse or RNase	- Compared to the control or RNase-treated ECV, PROse-treated ECV enhanced the migration of fibroblasts after 24 h treatment. - ECV or PROse-treated ECV increased cell index value of fibroblasts. RNase-treated ECV lacked this effect.	(37)
	CM	- After damage of hDFs by H_2_O_2_, hAEC-, and hAMSC-CM improved proliferation. - The $\frac{1}{2}$ conc. of hAEC-, but not hAMSC-, CM also improved proliferation. - All test groups markedly improved cell viability, with the hAEC-CM showing the strongest effect. - Both CMs improved normal hDFs migration in a scratch assay at 24 h, while only hAMSC-CM improved migration at 12 h. - hAEC-CM, but not hAMSC-CM, increased the percentage of cells in the S phase in comparison to damaged control. - Both CMs decreased the percentage of senescent cells in comparison to damaged controls (SA-β-gal). - Both CMs reduced reactive oxygen species production (ROS), increased activity of superoxide dismutase and catalase and decreased malondialdehyde and 8-OHdG levels (ELISA) in comparison to damaged hDFs. - Both CMs decreased γ-H2AX/H2AX ratio compared to damaged cells and lower ratio was achieved with hAEC-CM compared to hAMSC-CM. - mRNA levels of p21 and protein levels of p21 and p16 were reduced after either CM treatments compared to damaged control.	(38)
Keratinocytes	CM	- Increased the migration in a wound scratch assay at 6 h, with complete healing at 18 h. - Increased the phosphorylation of ERK, JNK, and AKT 15 min after treatment, which peaked at 60 min.	
		- Keratinocyte migration was completely blocked when cells were pre-treated with mitomycin C and ERK inhibitor (PD98059) or JNK inhibitor (SP600125), while it was not completely inhibited by AKT inhibitor (LY294002). Incubation with mitomycin C was to exclude their effect on cell proliferation. - Coculture with hAEC increased the expression of Cyclin D1, Cyclin D3, and Mdm2 as shown by Western blotting. - Coculture hAEC resulted in a higher S-phase fraction. - Compared with CM from fibroblasts, caused higher expression of the genes EGF, KGF, PDGF, CXCL-5, and SDF1, and lower expression of TGF-β1, while compared with CM from hAMSC, resulted in higher expression of the genes EGF, KGF, and CXCL-5 and lower expression of the genes PDGF, TGF-β1, and SDF-1.	(39)
Mouse BM-Neutrophils	ECV	- Treatment with either 1 or 5 μg of hAEC-ECV or 1 μg of hLF-ECV resulted in lower myeloperoxidase activity and higher cell death than controls.	(16)
Mouse T-lymphocytes (from spleen)		- Proliferation was suppressed by hAEC-ECV in comparison to hLF-ECV and control. - Using Foxp3-GFP knock-in CD4+cells and labeled exosomes with exo-red dye showed that uptake of hAEC-ECV corresponded to the maturation of CD4+ T cells into Foxp3-expressing cells, suggesting their Treg nature.	
Mouse BASCs	ECV	- Differentiated to alveolar, bronchiolar, and bronchioalveolar colonies after 21 days. - More and larger-sized bronchiolar and alveolar colonies resulted compared to medium-only control. - Higher expression of club cell marker (CC10) in bronchiolar colonies, and ciliogenesis marker Foxj1 in mixed colonies with ECV (5 and 10 μg) compared to control.	(16)
Human lung fibroblasts	ECV	- Exposure of HLF treated with TGF-β to ECV decreased myofibroblast differentiation in a dose-dependent way.	(16)
Mouse mesencephalic neuronal-glial cells	CM	- Pro-survival effect on lesioned neurons (treated with MPP+) in the form of increased longest neurite length and number of branching points per dopaminergic neuron. - Neutralizing antibodies against BDNF, CNTF, GMCSF, OSM, or NrCAM reduced the survival of dopaminergic neurons, the neurite outgrowth, the longest neurite length, and branching points per neuron, while neutralizing antibody against IL-1ra reduced the survival and branching points per neuron in CM-treated lesioned neurons.	(25)
Rat Embryonic (E18) cortical neurons	CM	- Remarkably Improved survival (91.9%).	(28)
Rat retinal ganglion cells	CM	- Enhanced their survival (52.3 ± 14.4%). The effect was superior to BDNF, CNTF, and NT-3. - This effect was retained only in the low-sized (<30 kDa) fraction of the CM.	(40)
Human umbilical cord blood-derived MSCs	CM	- Increased the number of DAT- and TH- positive cells. DAT and TH are considered dopaminergic neuron-specific markers. - Addition of K252a (Trk receptor inhibitor of BDNF and NGF) incompletely, but significantly, reversed this effect.	(29)
Primary mouse microglia	CM	- Decreased the CD86+ M1 subtype after LPS stimulation for 24 h as shown by FACs. - Decreased the number of apoptotic microglia and increased their phagocytic activity.	(41)
Human peripheral blood monocytes	Trans-well and CM	- Alteration of monocyte differentiation toward dendritic cells, resulting in cells that might be at an intermediate state of differentiation/maturation. The suppressive effect was less prominent compared to hAMSC.	(42)
Mouse peritoneal macrophages	CM	- Inhibited macrophage migration toward MIP-2. - Did not induce apoptosis (Caspase-3) or toxicity (trypan blue exclusion).	(43)
Mouse peritoneal neutrophils	CM	- Inhibited neutrophil migration toward MIP-2. - Did not induce apoptosis (Caspase-3) or toxicity (trypan blue exclusion).	(43)
Mouse lymphocytes	CM	- Decreased mouse T and B lymphocyte proliferation after mitogenic stimulation, but not without stimulation. - Induced apoptosis of T and B lymphocytes (Caspase-3 assay), 50% inhibition of this apoptosis was achieved with anit-FasL antibody.	(43)
Human CD4+ T-lymphocytes	ECV	- Inhibited lymphocyte proliferation (CSFE) with a more evident inhibition in case of small-sized ECV compared to large-sized ECV.	(44)
Human PBL	CM	- Inhibited lymphocyte proliferation after their exposure to either human pancreatic islets or PHA (mitogen).	(45)
Human fetal osteoblast cell line (hFOB1.19)	CM	- Increased proliferation, migration, and osteogenic differentiation (ALP, OCN, OPN, and RUNX2). - Neutralizing TGFβ1 antibody treatment attenuated the positive effect of CM on migration and osteogenic differentiation. - miR-34a-5p inhibitor transfection to hAEC decreased the hAEC-CM pro-differentiation effect.	(46)
Human aortic endothelial cells	CM	- Increased cell migration in scratch and trans-well migration assays. - Increased number of branching points at 6 and 48 h in Matrigel network formation assay.	(47)

*BASCs, broncho-alveolar stem cells; BM, bone marrow; BrdU, 5-bromo-2′-deoxyuridine; CM, conditioned medium; DAT, dopamine transporter; ECV, extracellular vesicle; EVDM, ECV-depleted conditioned medium; GO, gene ontology; hAEC, human amniotic epithelial cells; hAMSC, human amniotic mesenchymal stem cells; hFB, human fibroblasts; hGL, human granulosa-lutein cells; hLF, human lung fibroblast; HSCs, hepatic stellate cells; hUVECs, human umbilical vein endothelial cells; KEGG, Kyoto Encyclopedia of Genes and Genomes; LPS, lipopolysaccharide; MSCs, mesenchymal stem cells; MIP2, macrophage inflammatory protein-2; PROse, proteinase K; SMA, smooth muscle actin; TH, tyrosine hydroxylase. Cell lines: HK-2, human proximal tubular cell line; KGN, human granulosa-like tumor cell line; LX2, human hepatic stellate cell line*.

**Table 3:** *In vivo* studies demonstrating the paracrine effect of hAECs.

**System**	**Animal model**	**Disease pathology**	**Test groups**	**Control**	**ECV isolation**	**Peak size**	**Injection method**	**Frequency**	**Significant findings**	**References**
Cornea	Male New Zealand white rabbits	Corneal alkaline injury	hAEC-CM	1) Vehicle 2) Saline	None		0.2 mL into the dorsal bulbar subconjunctival using a tuberculin syringe with 26-gauge needle	On days 0, 1, and 2, and every other day thereafter	The sum of epithelial defect areas measured on day 6 and day 14 after injury in CM group was significantly smaller than those of vehicle, but not saline, groups.	(48)
Cornea	Male BALB/c mice	Neo-vascularization and MHC II APC recruitment	hAEC-CM	1) No treatment 2) DMEM/FCS 3) Recombinant human IL-1ra	None		A 5-μl drop of topical preparation	Three times a day for 2 weeks after corneal manipulation	CM significantly inhibited neovascularization (average inhibition = 22%) and MHC II APC recruitment when compared to the non-treated and placebo groups.	(49)
Kidney	Male C57BL/6j mice	Ischemia-reperfusion injury-induced Acute kidney injury	1) 1 ×10^6^ hAECs 2) 3 ×10^8^ hAECs-ECV	Vehicle (PBS)	DC	50–150 nm	100 μL injected into injured mice intravenously	At the end of the induction procedure	Both hAEC and ECV decreased mortality, serum creatinine, apoptosis (TUNEL) and increased mRNA levels of the angiogenesis-related genes (Fgf, Hgf, Igf-1, Pdgf, and Vegf) after surgery in comparison to vehicle group	(15)
Ovary	Female C57BL/6 mice	Chemotherapy induced premature ovarian failure	1) 4 ×10^6^ hAEC 2) hAEC-CM produced by 4 ×10^6^ hAEC	1) No POF 2) No treatment	None		IP transplantation (total volume of 0.2 mL)	24 h or 7 days after chemo-therapy (injection was repeated on the next day)	In comparison to chemo-damaged control: **hAEC-CM:** - Increased primordial follicles (24 h) - Increased antral follicles (24 h or 7 days) - Increased VEGFR1 and VEGFR2 (7 days) - Reduced VEGFA expression (24 h). **hAEC:** - Increased primordial and primary follicles (24 h). - Increased VEGFR1 and VEGFR2 expression (24 h and 7 days of chemotherapy, respectively). - Reduced VEGFA expression (24 h or 7 days). **Both treatments**: - Improved fertility (24 h). - Increased micro-vessel density (at 1 month of chemotherapy) CM>hAEC.	(50)
Ovary	Female C57BL/6 mice	Chemotherapy induced premature ovarian failure	1) 2 ×10^4^ hAEC 2) hAEC-CM produced by 2 ×10^4^ hAEC	1) No POF 2) Contra-lateral ovary (injected with vehicle DMEM/F12 in CM mice)	None		Volume of 10 μl injected into one of the ovaries of chemotherapy-induced POF using microinjection needle at laparotomy	1 week after the injection of chemo-therapy	In comparison to chemo-damaged control: **hAEC-CM:** - Increased primordial follicles. - Increased the expression of anti-Mullerian hormone, AMH, mouse vasa homolog, MVH, ovarian bone morphogenetic protein 15, BMP15 and hyaluronic acid synthase 2, HAS2. **hAEC:**	
									- Increased the expression of MVH **Both treatments**: - increased the number of secondary and mature follicles. - Increased micro-vessel density.	(6)
Ovary	Female C57BL/6 mice	Chemotherapy induced premature ovarian failure	1) hAEC-ECV 2)hAEC-CM 3) hAEC-CM without ECV	1) No POF 2) PBS	Total exosome isolation reagent (Invitrogen)	50–150 nm	Injected at 1 week (into ovaries via microinjection needle, 10 μL) and 2 weeks (tail vein injection, 100 μL) after chemotherapy		In comparison to chemo-damaged control: **hAEC-ECV:** - Increased the number of primordial, primary, and mature follicle. - Reversed the decreased biological processes induced by chemotherapy including brown fat cell differentiation, lipid catabolic process, metabolic pathways, proliferator-activated receptor gamma (PPAR?) and AMP-activated protein kinase (AMPK) signaling pathways, while it decreased other processes including cellular response to interleukin-1 and tumor necrosis factor. - Inhibited the acute vascular injury. - Decreased apoptosis of cumulus granulosa cells. - Upregulated hyaluronic acid synthase 2 expression. - Reversed the increased cleaved Caspase 3. **Both hAEC-ECV and CM**: - Increased the number mature follicles.	(19)
Ovary	Female B6AF1 mice	Autoimmune ovarian disease	1) 2 ×10^6^ hAECs 2) hAEC-CM	1) No AOD 2) No treatment AOD	None		200 μl were injected i.v. through the tail vein	at 3 time-points	In comparison to AOD control: Both treatments: - Increased E2 and decreased FSH and AZPAb serum levels. - Increased the number of primordial and mature follicles. - Decreased apoptosis marker Caspase-3 and fibrosis marker α-SMA. - Increased expression of M2 marker genes (CD206, FIZZ, and Arg-1). - Increased the percentage of Treg cells (CD4+CD25+Foxp3+) in the spleen.	(7)
Liver	Male C57Bl/6J mice	Liver fibrosis model (CCL_4_)	1) hAEC-CM 2) EVDM (≈ 2 ×10^6^ particles) 3) EV (≈ 24 ×10^6^ particles)	1) No CL4 2) Vehicle (saline)	DC	Mean 133.1 nm diameter	350 μL i.v.	3 times per week for the last 4 weeks of the study.	In comparison to CCL_4_ control: **hAEC-ECV:** - Lowered TGF-β in liver lysates **All treatments**: - Decreased liver fibrosis and α-SMA+ cells. - Decreased liver infiltrating macrophages (F4/80+) - Increased in M2 marker (CD206)	(17)
Liver	Male C57Bl/6J mice	Non-alcoholic steatohepatitis model (fast-food diet)	1) 2 ×10^6^ hAEC once 2) 2 ×10^6^ hAEC twice 3) 400 μL hAEC-CM	1) No NASH 2) No treatment NASH	None		i.p. injection	- hAEC: week 34 ± week 38. hAEC-CM: from week 34, 3 times per week for 8 weeks.	In comparison to NASH control: **hAEC-CM:** - Increased MMP-9 expression **All treatments**: - Reduced liver fibrosis area. - Reduced pSMAD 2/3 signaling (TGF-β1 signaling pathway) - **Reduced** activated hepatic stellate cells and liver macrophages.	(51)
Liver	Male C57Bl/6J mice	Liver fibrosis model (CCL_4_)	1) 2 ×10^6^ hAEC once 2) hAEC-CM	1) No CCL4 2) No treatment 3) DMEM/F12	None		- hAEC: in 200 μL of normal saline. - CM: 350 μL - i.v. through tail vein	hAEC: Once, 8 weeks after induction. hAEC-CM: from week 8, 3 times per week till week 12.	In comparison to their respective control: **hAEC** - Reduced A6+ and Pan-CK+ Liver progenitor cells (LPC) by twofold and threefold, respectively. **hAEC-CM:** - Reduced ALT, (2.4 folds) and AST, (3.6 folds) - Reduced the number of α-SMA+ cells. **Both treatments**: - Reduced liver fibrosis area.	(5)
Wound healing	Male C57Bl/6J mice	Full thickness wound 1 ×1 cm	1) hAEC-CM 2) CM+ERK inhibitor 3) CM+JNK inhibitor 4) CM+AKT inhibit	PBS control	None		−100 μL Injected at 4 sites surrounding the wound. - Inhibitor conc. (10 μM).	Day 1 and 3.	- The wound closure rate was higher on day 3 compared to control. - Wound area was smaller at 2 weeks compared to control. - Addition of ERK, JNK or AKT inhibitors impaired the organization of collagen	(39)
Wound healing	Male Sprague–Dawley rats	Full thickness wound 1 ×1 cm (4 wounds per rat)	1) 25 μg/mL ECV 2) 50 μg/mL ECV 3)100 μg/mL ECV	PBS	DC	50–150 nm	100 μL Injected at multiple sites surrounding the wound (1 wound per treatment per rat).	Once	No significant findings	(36)
Wound healing	Male Balb/c mice	Full thickness wound 1 ×1 cm	1) 50 μg/mL ECV 2) 50 μg/mL ECV + PROse 3) 50 μg/mL ECV + RNase	PBS	DC	30–150 nm (avg = 103 nm)	−100 μL Injected at 4 sites surrounding the wound. - Inhibitor conc. (10 μM).	Day 1 and 3.	- PROse-treated hAEC-ECV and hAEC-ECV treatment promoted the wound closure compared to the PBS control or the RNase-treated hAEC-ECV group on day 7.	(37)
Diabetic wound healing	db/db mice	Full thickness skin defect 0.8 cm	1) ECV (1,000 μg/ml) + DMSO 2) ECV + LY294002	PBS + DMSO	DC	Mean = 105.89 ± 10.36 nm	S.C injection	–	- Faster wound healing. - Higher thickness compared to control - These effects were dampened by LY294002 co-administration.	(23)
Lung	C57Bl/6 mice: 1) 6–8 weeks 2)12 months (aged mice)	Bleomycin model of lung fibrosis	1) 10 μg hAEC-ECV 2) 10 μg hLF-ECV	Saline	DC	80–120 nm	−100 μL i.v. injection - Aged mice: - Intranasal	- Day 1 (early) or Day 7 (late) after bleomycin. - Aged mice: - Day 7	Day 1 (early): - Reduction of CD4+ T cells and neutrophils in the spleen, and percentage of CD4+ T cells and interstitial macrophages in the lung. - Increased expression of β-catenin, BMP4, BMPR1a, and NFATC1 - BASCs showed significant reduction of c-MYC transcription, - Upregulation of Axin 1 and Axin 2 in MECs. - FGFR1 was elevated. Day 7 (late): - Tissue-to-airspace ratio was improved - Lung collagen content and αSMA expression were reduced - Number of BASCs per terminal bronchiole was higher. - Increased percentage of ATII - FZD6 expression was increased. Aged mice: - Reduction in αSMA^+^ cells. - BASCs per terminal bronchiole, percentage of alveolar type I and II cells increased.	(16)
Lung	Female Balb/c mice	1) Bleomycin-induced fibrosis 2) Chronic allergic airway disease (AAD)	1) 5 μg ECV 2) 5 μg ECV+RLX (0.5 mg/kg/day) 3) 25 μg ECV 4) 25 μg ECV+ RLX 5) 1 ×10^6^ AECs+ RLX 6) Pirfenidone (100 mg/kg/day, bid) in BLM only. 7) Historical RLX alone 8) No model induction		DC	–	- hAEC-ECV or hAEC: - Intranasally - RLX: S.C osmotic pump - Pirfenidone: - Oral gavage	For 7 days	- ECV Reduced interstitial inflammation in BLM. - ECV decreased airway inflammation in AAD model. - ECV with serelaxin was superior to hAEC with serelaxin in improving inflammation and reducing neutrophil and macrophage infiltration.	
									- ECV (5 or 25 μg) normalized the increased airway epithelial thickening in AAD model and the increased interstitial lung fibrosis in the BLM model. - Serelaxin + 25 μg ECV and serelaxin + hAEC groups normalized the subepithelial ECM thickness in AAD and BLM model, and total lung collagen concentration in AAD model. - ECV alone (at 5 or 25 μg) partly reduced airway TSLP-associated epithelial damage, epithelial TGF-β1 expression, and subepithelial myofibroblast accumulation in the AAD model and induced myofibroblast accumulation in the BLM model. - 25-μg ECV partly reduced BLM-induced epithelial damage. - Serelaxin augmented the ability of ECV to reduce chronic AAD-induced goblet cell metaplasia, epithelial TGF-β1 expression, and subepithelial myofibroblast differentiation and reduced BLM-induced lung epithelial damage and interstitial TGF-β1 expression. - Combining serelaxin and 25-μg ECV normalized chronic AAD-and BLM-induced airway/lung epithelial damage. - Combining serelaxin with 25-μg ECV had a stronger influence in reducing the AAD- or BLM- induced lung remodeling compared to serelaxin + 1 ×10^6^ hAEC.	(52)

*AAD, allergic airway disease; ALT, Alanine aminotransferase; AMH, anti-Mullerian hormone; AOD, autoimmune ovarian disease; APC, antigen presenting cells; AST, Aspartate aminotransferase; AZPab, anti-Zona Pellucida antibody; BASCs, broncho-alveolar stem cells; BLM, bleomycin-induced lung fibrosis model; BMP, bone morphogenetic protein; CCL4, carbon tetrachloride; CM, conditioned medium; DC, differential centrifugation, DMSO, dimethyl sulfoxide; ECV, extracellular vesicles; EVDM, ECV-depleted conditioned medium; FCS, fetal calf serum; FSH, follicle stimulating hormone; hAEC, human amniotic epithelial cells; HAS2, hyaluronic acid synthase 2; i.p., intraperitoneal; i.v., intravenous; MHC, major histocompatibility; MVH, mouse vasa homolog; NASH, non-alcoholic steatohepatitis; POF, premature ovarian failure; PROse, proteinase K; RLX, serelaxin; SMA, smooth muscle actin; Treg, T-regulatory lymphocytes*.

### *In vitro* Studies

#### Effect on Apoptosis

The effects of HAECs on apoptosis, as well as cell proliferation and angiogenesis seem to depend on the target cell/tissue type. HAECs were demonstrated to have a proapoptotic influence on tumor cells (53) and immune cells (43). The injection of HAECs into growing tumors diminished their final size, increased proapoptotic signaling markers, and reduced Bcl-protein levels (53). This effect was shown to be retained in the HAEC culture supernatant (53). These findings, together with the anti-proliferative and anti-angiogenic effects of HAECs on tumor tissue have proposed HAECs as an anti-tumor therapeutic strategy, as reviewed in (54). The variable effect on cell apoptosis was demonstrated when the immunomodulatory effects of HAECs were investigated. Although HAEC-CM had a proapoptotic effect on T and B lymphocytes, as shown by the Caspase-3 assay, a similar effect was not found in corneal epithelial cells or liver cells (43). Furthermore, macrophages and neutrophils were resistant to apoptosis induction by HAEC-CM (43).

Contrary to this, HAEC-ECV and CM protected other cell types from injury-induced apoptosis. HAEC-ECV protected HK-2 cells (immortalized human proximal tubular cells) from hypoxia-reoxygenation injury-induced apoptosis, as shown by Western blotting (cleaved Caspase 3) (15). When primary human granulosa-lutein cells (hGL) were cocultured with hAECs using a Boyden chamber, cleavage of Caspase 3 expression was significantly decreased in comparison to chemotherapy damaged control (6). The cocultured-hGL also showed significantly higher expression of P-SMAD2 and P-SMAD3 proteins in comparison to the chemotherapy-treated group suggesting activation of the Smad pathway through paracrine signals (6). The anti-apoptotic protective effect was also demonstrated in human ovarian granulosa tumor-derived cell line (KGN cells) (19), and the uptake of hAEC-ECVs by KGN cells was evidenced by fluorescent dye labeling. HAEC-ECVs significantly inhibited chemotherapy-induced granulosa cell apoptosis; shown by decreasing Bax and cleaved Caspase 3, increased anti-apoptotic genes, and Bad and Bcl2 expression. A similar reversal of chemotherapy-induced cleaved Caspase 3 elevation was seen when granulosa cells were transfected with miR-1246 or miR-21-5p mimic, which were identified in the hAEC-ECVs. Treatment with hAEC-ECVs resulted in a slight increase of miR-1246 in granulosa cells (qRT-PCR) highlighting a probable mechanism of action.

#### Cell Proliferation

In a similar fashion, the reported effects of hAECs on cell proliferation were also target-cell dependent. In four tumor cell lines, rat AEC-CM significantly inhibited proliferation to a variable degree (55). The effect was most prominent in the HepG2 cell line which showed a dose-dependent response, while non-tumorigenic mouse embryonic fibroblasts were resistant to this anti-proliferative effect (55). Another study showed that hAEC-CM inhibited the growth of epithelial ovarian cancer cell lines in a dose-dependent manner (30). A TGF-β1 mediated cycle arrest (G0/G1) was proposed as the underlying mechanism (30). hAEC+SK-OV-3 cancer cell line co-implantation in nude mice also resulted in a decrease in tumor size and weight (30). As a part of its immunomodulatory effect, hAEC-CM significantly inhibited T and B lymphocyte proliferation after mitogenic stimulation (43). In addition, hAEC-CM had a negative influence on hepatic stellate cells (HSCs) proliferation, as evidenced by Bromodeoxyuridine (BrdU) and platelet-derived growth factor-β expression (31). No effect on proliferation was observed in the granulosa cells (KGN cells) as shown by Western blotting (PCNA) (19).

On the other hand, HAEC-secretome components were frequently shown to promote cell proliferation in different responder cell types. The addition of hAEC-ECVs improved the proliferation of HK-2 cells (PCNA) (15). Hodge et al. (5) showed that hAEC-CM increased BrdU incorporation in liver progenitor cell (LPC) cultures (after 3 days of treatment) in comparison to untreated LPC. A similar effect on LPC culture was observed when they were treated with hepatocyte differentiation media. The KEGG pathway analysis of microarray results showed that genes associated with drug, glutathione, and arachidonic acid metabolism, steroid biosynthesis, and bile acid secretion were upregulated in LPC cultures treated with hAEC-CM. Interestingly, genes associated with cell proliferation and phosphoinositide 3-kinase signaling were downregulated. The GO pathway analysis showed that the upregulated genes were significantly enriched in multiple metabolic processes, negative regulation of cell growth, and apoptosis, while downregulated genes were enriched in pathways related to cell proliferation. In addition, the effect of CM on LPC proliferation *in vitro* was opposite to that observed *in vivo* (as described later). This was suggested by the authors to result from the dominance of the indirect (immunosuppressive effect) of CM over the direct effect on LPC, in addition to increased differentiation of LPC (5). Coculture of hAECs with keratinocytes through trans-well setting significantly increased the expression of Cyclin D1, Cyclin D3, (that are related to cell survival and proliferation), and Mdm2 as shown by Western blotting (39). Coculture also resulted in a higher S-phase fraction in comparison to control. The specific inhibition of the Akt pathway with inhibitor significantly decreased the CM-mediated stimulation of DNA synthesis (S-phase fraction) and cell proliferation. qPCR analysis showed that, compared with CM from fibroblasts, hAEC-CM caused higher expression of the genes EGF, KGF, PDGF, CXCL-5, and SDF1 and lower expression of TGF-β1, while compared with CM from human amnion mesenchymal stem cells (hAMSCs), hAEC-CM resulted in a higher expression of the genes EGF, KGF, and CXCL-5 and a lower expression of the genes PDGF, TGF-β1, and SDF-1. hAEC-CM significantly increased the proliferation of human fetal osteoblast cell line (hFOB1.19) cells (46). The effect of miR-34a-5p was examined, as it showed higher expression in hAECs in comparison to hAMSCs and hFOB1.19 cells. After transfection of hAEC with Cy3-conjugated miR-34a-5p mimics. The fluorescence signal was detected in hFOB1.19 cells, suggesting its transfer through the CM into the adjacent cells. Transfecting hFOB1.19 with the mimics resulted in inhibiting cell proliferation. CM from hAECs transfected with miR-34a-5p inhibitor resulted in attenuation of the pro-differentiation effect of CM.

Human amniotic epithelial cells-extracellular vesicles (hAEC-ECVs) also significantly increased the proliferation of human fibroblasts (hFBs) (23). The use of LY294002 to selectively block the PI3K-AKT-mTOR pathway significantly weakened this effect. The effects of 25, 50, or 100 μg/ml hAECs-ECV in 100 μl volume on human dermal fibroblasts (hDFs) in comparison to PBS were investigated (36). The study showed that hAEC-ECVs significantly enhanced the proliferation of fibroblasts. hAEC-ECVs or proteinase K (PROse)-treated hAEC-ECVs significantly increased cell index value of fibroblasts. However, RNase-treated hAEC-ECVs lacked this effect. This study highlighted the important role of ECV-miRNAs in wound healing promotion (37).

Furthermore, hAEC-CM had a protective effect on hDFs against hydrogen peroxide (H_2_O_2_)-induced senescence (38). The CM from hAECs was compared to CM from hAMSCs. Two concentrations were used for each CM (CM and its 1:1 dilution with growth medium resulting in 4 test groups). Results showed that after damaging hDFs by 200 μM H_2_O_2_, hAECs and hAMSC-CM significantly improved proliferation in comparison to control. The half concentration of hAEC-CM, but not hAMSC-CM, also significantly improved proliferation. All test groups showed a markedly improved cell viability (FDA staining) compared to damaged control, with the hAEC-CM showing the strongest effect. hAEC-CM, but not hAMSC-CM, significantly increased the percentage of cells in the S phase in comparison to damaged control as examined by flow cytometry. Another protective effect was demonstrated when several features of chemotherapy-induced damage in hGL cells were significantly reversed by hAEC coculture (Boyden chamber) including improved cell count (CCK-8 assay) and decreased Annexin-V^+^/PI^+^ cells (6).

This clearly-evidenced variation in response according to the target cells in regard to apoptosis and cell proliferation is difficult to explain since it variates from one effect to the opposite one, rather than the absence of effects.

#### Cell Differentiation

Data regarding the effect of hAEC-secretome on cell differentiation are relatively scarce. hAEC-CM was shown to induce differentiation of LPC toward hepatocytes after 8 days of culture, as demonstrated by the significant increase in alpha-fetoprotein (AFP) and albumin expression (5- and 8-fold, respectively). Interestingly, in the same study, hAEC coculture did not result in a significant increase of AFP or Albumin expression. The activity of tyrosine aminotransferase promoter, which is only active after differentiation into mature hepatocytes, was only observed in hAEC-CM treated LPC, as shown by X-gal staining. Urea production in the CM-treated group was significantly higher than untreated and hepatocyte-differentiation-medium-treated cultures after 16 days. Glycogen storage was also evidenced in the CM-treated group using Periodic acid–Schiff staining (5).

Cultured bronchoalveolar stem cells (BASCs) from normal mice differentiated to alveolar, bronchiolar, and bronchioalveolar colonies after 21 days when supplemented with hAEC-ECVs (16). Significantly more and larger-sized bronchiolar and alveolar colonies resulted compared to medium-only control. Significantly higher expression of club cell marker (CC10) in bronchiolar colonies and ciliogenesis marker Foxj1 in mixed colonies was reported with hAEC-ECV supplementation (5 and 10 μg) compared to control.

Regarding neurogenic differentiation, hAEC-CM significantly increased the number of dopamine transporter (DAT) and tyrosine hydroxylase (TH)- positive cells in human umbilical cord blood-derived MSCs culture, as shown by immunofluorescence (29). DAT and TH are considered dopaminergic neuron-specific markers. The addition of K252a (the Trk receptor inhibitor of BDNF and NGF) to CM-treated cultures significantly decreased the number of DAT- and TH-positive cells compared to the CM-treated group; however, the number was still significantly higher than the control group, implying incomplete blockage of action (29).

Influence on osteogenic differentiation was also reported (46). In this study, HAEC-CM significantly increased the expression of ALP, OCN, OPN, and RUNX2 in a human fetal osteoblast cell line (hFOB1.19 cells) compared to the control, which are markers of osteogenic differentiation. TGFβ1 was significantly higher in hAEC-CM as compared to the control medium. The addition of recombinant human TGFβ1 resulted in a significant increase in migration and expression of osteogenic differentiation markers of hFOB1.19 cells (ALP and RUNX2). Depletion of TGFβ1 in CM attenuated the effect of CM on osteogenic differentiation. To elucidate the mechanism, hFOB1.19 was transfected with miR-34a-5p mimics, which significantly enhanced osteogenic differentiation. More studies are required to understand the mechanism through which hAEC-secretome components influence cell differentiation.

#### Cell Migration

The effect of hAEC-secretome on cell migration is integral to its influence on angiogenesis, immune-modulation, and wound healing. When examining cell migration, the use of an appropriate assay and reasonable interpretation is necessary to exclude the influence of proliferative changes (56). In wound healing, a wound scratch assay showed that hAEC-CM significantly increased the migration of keratinocytes in comparison to control at 6 h, with complete healing at 18 h (39). hAEC-CM significantly increased the phosphorylation of ERK, JNK, and AKT 15 min after treatment, which peaked at 60 min. On the other hand, p38 phosphorylation showed no significant change. Keratinocyte migration was blocked when cells were pre-treated with ERK inhibitor (PD98059) or JNK inhibitor (SP600125), while it was not completely inhibited by AKT inhibitor (LY294002). Both hAEC-CM and hAMSC-CM significantly improved normal hDFs migration in a scratch assay at 24 h, while only hAMSC-CM significantly improved migration at 12 h (38). hAEC-ECVs remarkably increased the migration of fibroblasts (hDF) in a scratch assay in a dose-dependent manner compared to control (36). Compared to the control or RNase-treated ECVs, proteinase k (PROse)-treated hAEC-ECVs enhanced the migration of fibroblasts after 24 h treatment, which demonstrates the influence of ECV-miRNA (37). hAEC-ECVs also significantly increased the migration of hFBs, as shown by both scratch and trans-well assays (23). This effect was significantly weakened with the use of LY294002 to selectively block the PI3K-AKT-mTOR pathway (23). Using trans-well assay, hAEC-CM was shown to significantly increase hFOB1.19 cell migration (46). Depletion of TGFβ1 in CM attenuated the effect of CM on hFOB1.19 migration. Interestingly, transfecting hFOB1.19 with miR-34a-5p mimics resulted in migration inhibition. As a part of its *in vitro* pro-angiogenic effect, hAEC-CM was reported to increase hUVEC migration in a scratch assay (26) and human aortic endothelial cell migration in scratch and trans-well assays (47). Contrary to these positive impacts on cell migration, a negative impact on macrophage and neutrophil chemotaxis was reported as discussed later.

#### Anti-fibrotic Effect

A large portion of the studies investigating hAECs is directed toward their *in vivo* anti-fibrotic effect—whether in the setting of liver fibrosis or wound healing. Since the *in vivo* process is a complex cascade of events involving direct effects on collagen secreting cells and indirect anti-inflammatory and immunomodulatory effects, *in vitro* studies are necessary to understand their relative contributions. A research group at Monash University (31) examined the effect of hAEC-CM on hepatic stellate cells (HSCs) as the main collagen-secreting cells in the liver. Forty-eight hours after incubation with CM, HSCs showed a significant reduction of myofibroblast markers α-SMA (qPCR). TGF-β1 expression (ELISA) and collagen production ([3H] proline incorporation) were also significantly reduced, while a 2.74-fold increase in matrix metalloproteinase-9 (MMP-9) gene expression was observed. As previously mentioned, hAEC-CM had a negative influence on HSCs proliferation (BrdU and platelet-derived growth factor-β expression) and increased their apoptosis by 11.8% (Annexin V: PI FC). Soluble human leukocyte antigene-G1 (HLA-G1), known to be secreted by hAECs, significantly decreased TGF-β1 and collagen production by HSCs. The collagen production decrease was, however, to a less extent in comparison to CM. In a report by Alhomrani et al. (17), collagen production by human hepatic stellate cells (LX2 cell line), measured using [3H] proline incorporation, was significantly decreased when they were treated with hAEC-ECV or hAEC-CM compared to controls. These findings demonstrate a direct anti-fibrotic effect of hAEC-secretome that is affected through changes in HSCs.

In the wound healing scenario, excessive collagen production is implicated in scar tissue pathogenesis. A study examined the effect of hAEC-CM on hDF (35). Results showed that α-SMA, Col-I, and Col-III induced by TGF-β1 (qPCR and WB) were significantly repressed in the TGFβ1+ CM co-treatment group compared with that in TGF-β1 alone group. The activation of fibroblasts by TGF-β1 is an important step during keloid formation. hAEC-CM showed similar results in fibroblasts derived from keloid by significantly attenuating the expression of α-SMA and collagen I and III (qPCR and WB). After TGF-β1 stimulation of hDF, hAEC-CM significantly increased expressions of MMP-1 and TIMP-1, but not MMP-2 and TIMP-2 (qPCR and WB), in comparison to TGF-β1 activated control. CM attenuated the TGF-β1-induced contraction of F-actin bundles and decreased their number. Furthermore, supernatant collagen level was reduced when keloid fibroblasts were treated with hAEC-CM as compared to control. On the other hand, MMP-1, MMP-2, and TIMP1 were significantly upregulated in keloid fibroblasts by CM. In order to understand the underlying mechanism, HLA-G contribution was investigated. HLA-G in levels like that of hAEC-CM significantly reduced Col-I, but not Col-III proteins, induced by TGF-β1. The reduction also did not reach the same level as achieved by hAEC-CM. Phospho-Smad2/3 export from the nucleus to the cytoplasm after the addition of CM or HLA-G supported their interference with TGF-β1 activation of fibroblasts. Another study examined the dose-dependent effects of hAEC-ECV on hDF (36). Western blotting showed that Col-I and Col-III were significantly repressed by 100 μg/ml ECV-treatment. This finding was augmented by immunofluorescence results. Collagen in the supernatant (Sircol collagen assay) was also significantly reduced by this ECV concentration. MMP-1 mRNA was significantly upregulated in this treatment group while TIMP-1 was only slightly increased. To understand the influence of hAEC-ECVs on lung fibrosis, human lung fibroblasts (hLFs) treated with TGF-β were supplemented hAEC-ECVs. This significantly decreased myofibroblast differentiation in a dose-dependent style. Although it also inhibited collagen production by hLFs, the inhibition was not statistically significant (16).

#### Angiogenesis

Perhaps the most controversial aspect of hAEC effect is that related to angiogenesis. This controversy emerged from reports suggesting either a pro-angiogenic or an anti-angiogenic effect of hAECs or their derivatives. The hAEC-CM contains a relatively high level of known angiogenic factors, as compared to CM from human aortic endothelial cells, including HGF, IGF-1, VEGF, EGF, HB-EGF, and bFGF (47). Secretion of angiogenic factors, including angiogenin (ANG), epidermal growth factor (EGF), and interleukin (IL)-6, was shown to increase by hypoxic culture conditions (27). In addition, most of the reported *in vitro* findings support a pro-angiogenic effect. hAEC-CM was shown to significantly promote hUVECs proliferation in a CCK-8 assay and migration in a trans-well assay (26). In another study (23), hAEC-ECVs significantly promoted the proliferation of hUVECs and increased the number of capillary-like structures formed by hUVECs (tube-formation assay). These effects were significantly diminished by the LY294002 addition. Similarly, hAEC-coculture significantly increased the mean tube length of hUVECs in tube formation assay (6). When the effects of CM from hAECs and hAMSCs on human aortic endothelial cells were compared (47), hAEC-CM significantly increased the cell migration in a scratch assay and trans-well migration assay compared to control, while hAMSC-CM significantly promoted the cell proliferation as shown by CCK8 assay and increased the cells in S-phase in cell-cycle analysis. Both CM resulted in a significantly higher number of branching points in a Matrigel network formation assay (at 48 and 6 h with hAEC-CM only) and resulted in blood vessel network formation in an *in vivo* Matrigel plug model in mice as compared to CM from aortic endothelial cells. Both CMs contained higher levels of angiogenic factors as compared to CM from human aortic endothelial cells. However, hAEC-CM contained a higher level of HB-EGF and a lower level of HGF and bFGF compared to hAMSC-CM.

On the other hand, hAECs were shown to express several anti-angiogenic substances including interleukin-1 receptor antagonist (IL-1ra), collagen XVIII (precursor of endostatin), thrombospondin-1, and all four TIMPs (57). Interestingly, one study using an aortic ring assay demonstrated an anti-angiogenic effect of amniotic membrane in the presence of its epithelial cell layer, while this effect was switched to a pro-angiogenic effect when the hAECs were removed from the amniotic membrane (53).

The controversy extends to the *in vivo* studies. In addition to the aforementioned Matrigel plug model, hAEC-CM injection in a premature ovarian failure model significantly increased VEGFR1 and VEGFR2 expression and reduced VEGFA expression (50). CM also significantly increased the micro-vessel density (CD34) in comparison to control after either intraperitoneal (50) or local injection (6). In an acute kidney injury model, hAEC-ECV treatment resulted in significant upregulation of angiogenesis-related genes (*Fgf, Hgf, Igf-1, Pdgf*, and *Vegf*) (15). On the other hand, topical application of hAEC-CM in a mouse model of corneal neovascularization significantly inhibited neovascularization (49). The utility of the rat dorsal skin chamber model showed that angiogenesis was promoted when the epithelial side of the membrane was facing up (away from the rat), while it was inhibited when the mesenchymal side was facing up. Thus, the authors suggested that the effect of the amniotic membrane is side-dependent (58). The same group hypothesized that the anti-angiogenic effect of the amniotic membrane is mediated through the inhibition of heat-shock protein-90 (59). It should be noted, however, that changing sides does not exclude the diffusion of soluble factors from the other membrane compartment. The use of similar quantifiable vascularization models with isolated hAECs or their derivatives is still necessary to understand their isolated *in vivo* effect.

In a trial to unravel this controversy, Zhu et al. (60) compared hAECs from term and preterm placentae. Both types of hAECs expressed VEGFA, PDGFB, ANGPT1, and FOXC1, which increased significantly with TNFα and IFNγ stimulation. The CM from term hAECs resulted in longer hUVEC tubules on Matrigel. *In vivo* assessment showed that intraperitoneal injection of term hAECs, but not preterm hAECs, decreased angiogenesis in a bleomycin lung fibrosis model, while it had a pro-angiogenic effect in a neonatal model of hyperoxia-induced lung injury. The study highlighted that the presence of inflammation, along with the underlying pathology, might contribute to the influence of hAECs on angiogenesis in the *in vivo* setting. As a note, it would be desirable to examine the *in vivo* angiogenic effects outside the reparative context.

#### Immunomodulatory Effects

The understanding of the immunomodulatory effects of hAEC-secretome *in vitro* is crucial as they were commonly reported to contribute to the results of the animal studies, as described later. Multiple studies examined the immunomodulatory effect of hAEC-secretome that mainly focused on the modulation of macrophage polarization. hAEC-ECV was shown to promote mouse bone marrow-derived macrophage polarization toward M2 phenotype as evidenced by a significant increase in M2 markers (CD206, CD163, IL4rα, and Arg1 mRNA) expression and downregulation of M1 markers (CD86, IFNγ, TNFα, and iNOS mRNA) after 7 days of culture (15). In another study, mouse RAW264.7 macrophages were treated with 1 μg/ml LPS and hAEC-CM was added at the same time with or without neutralizing TGF-β antibody (7). After 24 h, LPS significantly increased the expression of pro-inflammatory genes (TNF-α, IL-6, IL-1β, and iNOS-2) and decreased the expression of anti-inflammatory genes (IL-10 and CD206). CM treatment significantly inhibited the pro-inflammatory markers TNF-α, IL-6, and iNOS-2 and increased the expression of IL-10 and CD206 genes. This effect was partially attenuated when an anti-TGF-β neutralizing antibody was added. Interestingly, in the absence of LPS, no significant effect of CM on these markers was noted. CM also significantly inhibited the migration of RAW264.7 cells that was induced by LPS in a macrophage migration assay. ELISA results showed that a high level of macrophage inflammatory factor (MIF) is secreted into hAEC-CM. A neutralizing antibody against MIF attenuated the effect of CM on macrophage migration assay.

Treatment of bone marrow-derived macrophages with either hAEC-ECVs, -CM, or -EVDM after 1 day of induction of M0 macrophages to either M1 (LPS + INFγ) or M2 (IL4 + IL13) phenotypes resulted in significantly increased M2 marker (CD206, FC) in the M1 induction group, while no significant influence was noted in the M2 induction group or naïve macrophages group after addition of the preparations (17).

Exposure of mouse bone marrow-derived macrophages to hAEC-CM, with or without pre-stimulation with LPS, significantly reduced the proportion of CD86+ cells and significantly increased the proportion of CD206+ cells (flow cytometry) (33). qPCR showed a modest increase in M2-specific genes in macrophages after LPS exposure, which was further increased when macrophages were cultured in hAEC-CM as compared to LPS-exposed control. hAEC-CM significantly reduced the chemotaxis of macrophages toward rMIP-2 and increased their phagocytic ability. On the other hand, CM had no significant effect on macrophage proliferation, regardless of exposure to LPS. These findings demonstrate that hAEC-CM can have a direct effect on macrophages which is independent of the other immune cell populations. In the same context, hAEC-CM significantly increased the M2 macrophage marker (CD206, WB) and decreased the M1 macrophage marker (iNos, WB) in primary mouse macrophage culture treated with TNF-α + INF-γ *in vitro* (26).

A study compared the ability of hAMSCs and hAECs to suppress the development and maturation of human peripheral blood monocyte-derived dendritic cells in response to IL-4 and granulocyte-macrophage colony-stimulating factor (GM-CSF), followed by LPS treatment (42). hAMSC, in a trans-well setting, showed more prominent inhibition of dendritic cell development in comparison to hAECs. Trans-well culture with hAECs resulted in alteration of monocyte differentiation toward dendritic cells, resulting in cells that might be at an intermediate state of differentiation/maturation. The effect of hAECs was generally less stable by increasing the passage used as compared to hAMSCs. Similar results to trans-well culture were obtained with CM from P0 of both cell types. After stimulation of primary mouse microglia with LPS for 24 h, the addition of hAEC-CM significantly decreased the CD86+ M1 subtype as shown by flow cytometry. CM also significantly decreased the number of apoptotic microglia and increased their phagocytic activity but did not impact their proliferation (41).

In another study (16), the effect of hAEC-ECVs was examined on different immune cell populations in comparison to ECV from human lung fibroblasts (hLF-ECVs). Neutrophils (isolated from C57Bl/6 mice bone marrow) treated with either 1 or 5 μg of hAEC-ECVs or 1 μg of hLF-ECVs showed significantly lower myeloperoxidase activity and higher cell death than controls. Macrophages (isolated from C57Bl/6 mice bone marrow) treated with 5 μg hAEC-ECVs showed double the phagocytic activity in comparison to control (effect of hLF was remarkably lower than hAEC-ECVs but not significant). ECVs treatment also significantly increased the percentage of CD206^+^ (M2 marker) and significantly lowered CD86^+^ (M1 marker) cells in comparison to control and hLF-ECVs. T-lymphocyte (CD4^+^ cells enriched from C57Bl/6 mice spleen) proliferation was also significantly suppressed by hAEC-ECVs in comparison to hLF-ECVs and control. Using Foxp3-GFP knock-in, CD4^+^cells and labeled exosomes with exo-red dye showed that uptake of hAEC-ECVs corresponded to the maturation of CD4+ T cells into Foxp3-expressing cells, suggesting their Treg nature, as evidenced by their co-localization.

Another group (43) showed that hAEC-CM significantly inhibited mouse neutrophil and macrophage migration toward macrophage inflammatory protein (MIP-2). The addition of CM significantly decreased mouse T and B lymphocyte proliferation after mitogenic stimulation as shown by the 3H-thymidine incorporation assay. No significant effect on lymphocyte proliferation was found when no mitogenic stimulation was performed. CM induced apoptosis of T and B lymphocytes (Caspase-3 assay), but not macrophages and neutrophils. CM was also not toxic to macrophages and neutrophils (trypan blue exclusion). A 50% reduction of lymphocyte apoptosis was achieved when an anti-FasL antibody was added, but not by anti-TRAIL or anti-TNF antibodies.

In a well-designed study (44), soluble isotypes of HLA-G and HLA-E were measured in the hAEC-CM using ELISA. The study showed that both are released (13.52 ± 1.16 ng/ml and 7.54 ± 0.57 ng/ml, respectively) after 24 h of culture and the amount increased after culturing for 2 more days (i.e., 72 h). Both soluble HLA-G1 and HLA-G5 were demonstrated in the CM in most of the batches. HLA-G was also found at high levels on large-size ECVs and to a less extent on small-size ECVs as shown by flow cytometry. Inhibitory effect of hAECs on CD4^+^ T cell proliferation was significantly reduced with pretreatment of hAECs with anti-HLA-G or anti-β2 microglobulin, and, to a lesser extent, in the presence of anti-HLA-E blocking antibody. The addition of small or large-sized ECV significantly inhibited T-cell proliferation, with a more evident inhibition with the small-sized ECV. In addition, pretreatment of hAECs with either specific inhibitors of small-sized ECVs (Manumycin A and GW4869) or large-sized ECV (D-Pantethine) significantly reduced their inhibitory effect on T-cell proliferation, although an evident inhibitory effect can still be noted. hAEC-CM also significantly reduced human peripheral-blood-lymphocyte proliferation after their exposure to human pancreatic islets (45). CM also significantly inhibited mitogen (phytohemagglutinin, PHA)-induced lymphocyte proliferation.

#### Others

The protective and reparative capacity of hAEC-secretome was demonstrated in multiple studies that utilized different forms of cell injury models. In a study (19) examining the effect of hAEC transplantation in a mouse intrauterine adhesion (IUA) model, the investigators examined the paracrine effect of hAEC when cocultured with human endometrial mesenchymal stem cells damaged by H_2_O_2_ using a trans-well insert. HAEC coculture resulted in improving the morphological damage resulting from H_2_O_2_. In addition, coculture significantly decreased p62 protein level (WB), an indicator of autophagic flux inhibition, increased the LC3-II/LC3-I ratio (WB) in comparison to damaged control and restored the levels of estrogen receptor (ER, WB), which was reduced by H_2_O_2_ treatment. The results showed that the positive effects of hAEC transplantation in the IUA model can be partly explained by autophagy induction in a paracrine fashion (19). A senescence-associated β-galactosidase (SA-β-gal) activity assay showed that both hAEC-CM and hAMSC-CM significantly decreased the percentage of senescent cells in comparison to damaged controls (38). Both CMs significantly reduced reactive oxygen species production (ROS), increased activity of superoxide dismutase and catalase, and decreased malondialdehyde and 8-OHdG levels (ELISA) in comparison to damaged hDF. Both also significantly decreased γ-H2AX/H2AX ratio compared to damaged cells and a significantly lower ratio was achieved with hAEC-CM compared to hAMSC-CM. mRNA levels of p21 and protein levels of p21 and p16 were significantly reduced after either CM treatments compared to damaged control. overexpression of p21 and p16 was previously reported to cause premature cell senescence (61, 62). Another study (25) examined the effect of hAEC-CM on primary mesencephalic neurons treated with MPP+ (an active metabolite of MPTP, that is selectively taken up by dopaminergic neurons resulting in toxicity). MPP+ induced damage was demonstrated by reducing the number of tyrosine hydroxylase (TH)-positive cells and increasing neurite fragmentation. hAEC-CM induced a pro-survival effect on lesioned neurons in the form of a significant increase in longest neurite length and number of branching points per dopaminergic neuron. In antibody neutralization experiments, the addition of neutralizing antibodies against BDNF, CNTF, GMCSF, OSM, or NrCAM significantly reduced the survival of dopaminergic neurons, the neurite outgrowth, the longest neurite length, and the branching points per neuron, while neutralizing antibody against IL-1ra significantly reduced the survival and branching points per neuron. No significant effect was noted with neutralizing antibodies against IL-10 and IL-13.

A pro-survival influence of hAEC-secretome was demonstrated when hAEC-CM significantly improved the survival of embryonic cortical neurons (28). This neurotrophic effect of hAEC-CM (91.9% survival) was superior to that of other neurotrophic factors including BDNF, NGF, bFGF, TGF-β1, and PDGF. EGF also significantly improved survival (54.3%), however, it was not detected in the CM by enzyme immunoassay, suggesting the presence of unidentified neurotrophic factors in hAEC-CM. The same suggestion was offered by another study that examined the effect of hAEC-CM on chicken neural retinal cells (32). CM improved neural cell survival and neurite-like growth in comparison to control (semi-quantitative). A similar study design (40) showed that hAEC-CM significantly enhanced the survival of rat retinal ganglion cells and was significantly superior to the effect of BDNF, CNTF, and NT-3. When the CM was separated into high and low-sized fractions (cut-off of 30 kDa), only the low-sized fraction significantly promoted the survival of retinal ganglion cells (40).

In a trial to examine the role of ECV from oxidatively stressed hAEC as a possible mediator for the initiation of labor and a tool for fetal-maternal biochemical communication, a study (34) examined the effect of ECV secreted by oxidatively stressed hAEC in inducing inflammatory markers in myometrial, decidual, and placental cells *in vitro*. Stressed hAEC secreted more exosomes per cell (1,211 vs. 899 in control, separated by differential centrifugation) and they were taken by the three cell types as evidenced by confocal microscopy. Treatment of cells with ECV derived from normal (control) or stressed hAEC significantly increased the secretion of IL-6, IL-8, and PGE2, but not IL-1β or TNF-α (ELISA, levels were only slightly higher in case of stressed-exosomes) and increased activation of NF-κβ (WB for total and phosphorylated NF-κβ) in myometrial and decidual cells. No effect was observed on the level of IL-6, IL-8, PGE2, IL-1β, or TNF-α in placental cells. The study also showed increased staining of NANOG, a stem cell marker that is also present in hAEC-ECV in myometrial and decidual tissues from samples taken at term delivery (IHC dual staining with CD9) in comparison to not-in-labor deliveries. Results showed that hAEC-ECV, secreted in the presence or absence of oxidative stress, causes inflammatory activation in myometrial and decidual cells but not placental cells *in vitro*. In another study (63), heat shock protein (HSP) 70 and activated form of pro-senescence and term parturition associated marker (P-p38) were significantly higher in ECV from hAECs that were subjected to oxidative stress.

### Effect in Experimental Animal Models (According to Pathology)

The effect of hAECs secretome was examined in several animal models of disease pathology. Translating *in vitro* findings to functional improvements in animal models is a crucial step for the progress of secretome preparations as a potential therapeutic strategy.

#### Cornea

Since the introduction of the fetal membrane as a tool for corneal repair by De Rotth (64), and the refinement of the approach to the use of amniotic membrane for corneal surface reconstruction by Kim and Tseng (65), the amniotic membrane has been extensively used for corneal repair in a myriad of clinical pathologies (66) mainly for its anti-inflammatory effect and its ability to promote corneal epithelial healing. These effects were hypothesized to be similarly affected by hAEC-secretome. hAEC-CM seemed to result in a better corneal epithelization after alkaline injury in the rabbit model as evidenced by the size of the defect, the frequency of intact epithelium, and the infiltration with inflammatory cells (48). The sum of epithelial defect areas in the CM group at day 6 and day 14 was significantly smaller than the control medium (keratocyte serum-free medium), but not the saline group.

The topical application of hAEC-CM on the cornea, in a mouse model of neovascularization and MHC II antigen-presenting cells recruitment, significantly inhibited neovascularization (average inhibition = 22%). The study showed that hAEC-CM contained 235.8 pg/ml of human interleukin-1 receptor antagonist (IL-1ra). However, applying the same concentration of IL-1ra resulted only in 11% inhibition of neovascularization. CM also significantly inhibited MHC II APC recruitment when compared to the non-treated and placebo groups. In addition, the expression of IL-1ra and IL-1β mRNA was markedly decreased in the CM-treated corneas. The study suggested the presence of anti-inflammatory mediators, other than IL-1ra, in the hAEC-CM (49). hAEC-derived topical preparations can be a convenient alternative or supplement for the well-established amniotic membrane corneal therapy. Therefore, more studies are required to demonstrate the potential of hAEC- secretome in this regard.

#### Kidney

Only one study examined the effect of hAEC-ECV in an acute kidney injury (AKI) model. The study compared the systemic administration of hAEC vs. hAEC-ECV in a mouse model of ischemic-reperfusion injury-induced acute kidney injury. Both hAEC and ECV administrations significantly decreased mortality, serum creatinine, number of apoptotic cells (TUNEL), and markedly increased tubular cell proliferation (Ki67 staining) and mRNA levels of the angiogenesis-related genes (*Fgf, Hgf, Igf-1, Pdgf*, and *Vegf*) after AKI in comparison to control group. Treatment groups showed higher differentiation of CD206^+^/F4/80^+^ M2 type macrophages, as shown by flow cytometry, higher anti-inflammatory (IL-4 and IL-13), and lower pro-inflammatory (IFNγ and TNFα) cytokine expressions (15).

#### Ovary

In a series of reports, a group of researchers investigated the effects of hAEC, hAEC-CM, and hAEC-ECV in animal models of chemotherapy-induced premature ovarian failure (POF). In the first study, they examined the effect of intraperitoneal injection of hAEC or hAEC-CM in the POF mouse model (50). Results showed that compared to the chemo-damaged ovaries, the number of primary and primordial follicles significantly increased in mice receiving hAEC-CM after 24 h, while antral follicles increased significantly in mice receiving CM treatment after 24 h or 7 days of chemotherapy. Fertility was also significantly improved by both hAEC and hAEC-CM treatments when installed 24 h after chemotherapy in comparison to chemo-damage control. Compared to chemo-damaged tissue, hAEC treatment significantly increased VEGFR1 and VEGFR2 expression when they were injected after 24 h and 7 days of chemotherapy, respectively, and reduced VEGFA expression when injected at either 24 h or 7 days. On the other hand, CM had a similar effect on VEGFR1 and VEGFR2 when injected at 7 days only, while it had a similar effect on VEGFA when injected at 24 h only. Micro-vessel density calculation (CD34 staining) showed a significant increase in all treatment groups in comparison to the chemotherapy group after 1 month of chemotherapy injection. At this time point, the density in the CM group was significantly higher than in the hAEC group. These results proposed the improvement of angiogenesis as a protective mechanism of hAEC treatment in the POF model. It can be concluded that hAEC-CM could reproduce the majority of hAEC transplantation effects in this model, although the magnitude was occasionally superior or inferior.

The same group compared the effect of direct Injection of hAEC or hAEC-CM into the ovary using the same animal model. This time (6), they examined the effect of hAEC or hAEC-CM injection into a unilateral ovary of the POF mouse model, keeping the other side as a control. Both treatments significantly increased the number of secondary and mature follicles, while only hAEC-CM significantly increased primordial follicles compared to the control side, as shown by histological examination. hAEC-CM significantly increased the expression of genes involved in follicle growth (anti-Mullerian hormone, AMH), primordial germ cells (mouse vasa homolog, MVH), ovarian folliculogenesis (bone morphogenetic protein 15, BMP15), and cumulus expansion (hyaluronic acid synthase 2, HAS2) in chemo-damaged ovaries in comparison to control samples (real time-PCR). MVH was also significantly increased by hAEC treatment. Both treatments significantly increased micro-vessel density in chemo-damaged ovaries as shown by the number of CD34-positive cells. These findings show that the protective effects in POF can be produced by directly mediated to the ovarian tissue after local administration of hAEC preparations.

The third study in the series (19) examined the effect of hAEC-ECVs on POF induced by chemotherapy in a mouse model. This showed that hAEC-CM significantly increased the number of mature follicles, while hAEC-ECVs significantly increased primordial, primary, and mature follicle count compared to the control group. mRNA array analysis showed that hAEC-ECV reversed the decreased biological processes induced by chemotherapy treatments. These processes included brown fat cell differentiation, lipid catabolic process, metabolic pathways, proliferator-activated receptor gamma (PPAR?), and AMP-activated protein kinase (AMPK) signaling pathways, while it decreased other processes including cellular response to interleukin-1 and tumor necrosis factor in the ovaries of POF mice highlighting the potential of hAEC-ECVs to repair ovarian function. hAEC-ECVs also significantly inhibited the acute vascular injury induced by chemotherapy. Although ECV did not increase granulosa cell proliferation (EdU), it significantly decreased chemotherapy-induced apoptosis of cumulus granulosa cells (TUNEL) which play a vital role in the regulation of cumulus-oocyte complexes, upregulated hyaluronic acid synthase-2 expression, which was significantly reduced by chemotherapy treatment, and reversed the increased cleaved Caspase 3 that was induced by chemotherapy.

The same group investigated the effect of hAECs and hAEC-CM in the autoimmune ovarian disease (AOD) mouse model (7). Both treatments were injected intravenously through the tail vein at 3 time-points in B6AF1 mice immunized with zona pellucida protein 3 peptides (pZP3). Both treatments regulated the disordered estrous cycle caused by pZP3 injection and partially, but significantly, increased E2 and decreased FSH and AZPAb serum levels in comparison to AOD control mice. Both treatments significantly increased the number of primordial and mature follicles, and decreased apoptosis marker Caspase-3 and fibrosis marker α-SMA compared to AOD controls. Both treatments increased M2 macrophages (CD68^+^CD163^+^) in AOD ovaries and significantly increased the expression of M2 marker genes (CD206, FIZZ, and Arg-1) in comparison to AOD control. The treatments also significantly increased the percentage of Treg cells (CD4^+^CD25^+^Foxp3^+^) in the spleen in comparison to AOD control, as shown by flow cytometry.

#### Liver

The positive effects of hAEC transplantation on liver fibrosis are demonstrated by several studies (2). On the other hand, few studies demonstrated the effect of hAEC-secretome on liver pathology models (5, 17, 51). A research group at Monash University examined the effect of hAEC-CM, hAEC-ECV, and hAEC-ECV-depleted-CM (EVDM) on liver fibrosis, hepatic stellate cells (HSCs), and macrophages in a mouse carbon tetrachloride (CCL_4_) liver fibrosis model (17). The three preparations were injected intravenously through the tail vein 3 times per week after 8 weeks of fibrosis induction by CCL_4_ until the end of the study (12 weeks). The study showed that the 3 preparations significantly decreased liver fibrosis (Picro-Sirius Red staining), and the number of α-smooth muscle actin (α-SMA)^+^ cells. Only hAEC-ECV treatment resulted in significantly lowered TGF-β, a potent pro-inflammatory cytokine, in liver lysates compared to the control group. All three preparations significantly decreased liver infiltrating macrophages (F4/80^+^). CCL_4_ treatment significantly increased the density of CD86^+^ macrophages (M1 marker), while injecting ECV, CM, or EVDM resulted in a significant increase in the density of CD206^+^ macrophages (M2 marker). The polarization of macrophages toward the M2 phenotype was previously reported to be associated with the resolution of liver fibrosis (67).

The same group (51) investigated the effect of hAEC-CM on another model of liver pathology, the non-alcoholic steatohepatitis (NASH) model, in mice. The mice received hAEC after 34 weeks of NASH induction (Group 1), an additional hAEC injection at week 38 (Group 2), or hAEC-CM intraperitoneally 3 times per week for 8 weeks starting from week 34 (Group 3). Although all three treatments did not significantly alter the NASH activity score or metabolic parameters such as body weight, total cholesterol, or glucose tolerance, they significantly reduced liver fibrosis area (Picro-Sirius Red), pSMAD 2/3 signaling (TGF-β1 signaling pathway), and activated hepatic stellate cells (α-SMA, IHC) and liver macrophages (F4/80 staining). Only the CM group significantly increased MMP-9 expression in comparison to the no-treatment group. However, gelatin zymology did not show significantly increased protein density.

In another study by the same group, the authors compared the effect of hAEC and hAEC-CM on liver fibrosis in the CCL_4_ mouse model (5). The hAECs were administered as a single injection while CM was injected 3 times weekly during the study period through the tail vein (i.v.). Mice receiving control medium served as a control for CM. There was no significant difference between the control groups and hAEC or hAEC-CM groups regarding Ki67 staining. hAEC significantly reduced A6^+^ and Pan-CK^+^ Liver progenitor cells (LPC) by 2- and 3-fold, respectively. Both CM and control medium significantly reduced LPC cells by 4.5- and 3.5-fold, respectively, without a significant difference between them. CM significantly reduced serum alanine aminotransferase (ALT, 2.4-fold) and aspartate aminotransferase (AST, 3.6-fold) in comparison to the control medium, while hAEC had no significant effect. Both hAEC and CM treatment significantly reduced liver fibrosis area by 33 and 34%, respectively, in comparison to their respective controls. Only CM significantly reduced the number of α-SMA^+^ cells (37%). Both treatments failed to significantly decrease the number of macrophages (F4/80^+^) per field that was increased in response to CCL_4_ treatment when compared to their respective control groups. These studies demonstrated that both hAEC-CM and -ECV possess an antifibrotic effect on the liver both in CCL_4_ and NASH models, which is mediated through a direct effect on HSCs and an indirect influence through shifting the macrophage polarization toward M2 phenotype. These findings were supported by the *in vitro* studies. The effect on LPC, however, was proposed to be of indirect nature as an *in vitro* study showed that hAEC-CM improved LPC proliferation (5). More research is necessary to identify the peptide or miRNA mediators of this effect.

#### Wound Healing

Clinical use of amniotic membrane for wound dressing was associated with favorable outcomes and was proposed as a cost-effective alternative to conventional dressings (68–70). Four studies examined the utility of hAEC-secretome components in full-thickness skin defect murine models. In the first study, Zhao et al. (39) injected hAEC-CM in a full-thickness skin defect mouse model into the surrounding tissues of the wound at four sites on days 1 and 3. The mice were followed up till day 14 for wound size and closure rate. The wound closure rate was significantly higher in the CM group compared to control on day 3 and complete epithelization was achieved on day 7. The wound area was also significantly smaller than that of controls at 2 weeks. Histological examination at 2 weeks showed that healing was faster in the CM group and that the addition of ERK, JNK, or AKT inhibitors impaired the organization of collagen.

The same group (36) investigated the effect of hAEC-ECV on wound healing in a full-thickness skin defect 1 cm ×1 cm in a rat model. One hundred microliters of PBS control, 25, 50, or 100 μg/ml ECV were subcutaneously injected around the wound at multiple points. ECV-treated wounds showed increased re-epithelialization rates in a dose-dependent manner in comparison to control. Histology also showed better collagen organization in ECV-treated wounds.

To further understand the influence of hAEC-ECV, the effect of purified hAEC-ECV together with either proteinase K (PROse) or rNase A on fibroblasts and cutaneous wound healing was investigated (37). One hundred microliters of PBS control or 50 μg/ml ECV or an equal amount of ECV together with either PROse or rNase A were injected at four sites into the surrounding tissue of a full-thickness skin defect in Balb/c mouse model of the wounds on day 1 and 3. PROse-treated hAEC-ECV and hAEC-ECV treatment significantly promoted the wound closure compared to the PBS control or the rNase-treated hAEC-ECV group on day 7. All wounds were completely healed by day 14 but PROse-treated hAEC-ECV and hAEC-ECV groups seemed to be flatter in comparison to the control group or rNase-treated hAEC-ECV group. This study suggested that the positive influence of ECV on wound healing is mainly mediated through the miRNA cargo, the identification of the possible miRNA candidates is further required.

A study by Wei et al. (23) examined the effect of hAEC-ECV on diabetic wound healing. Full-thickness skin wounds in diabetic (db/db) mice were used for this evaluation. Faster wound healing was noted after subcutaneous ECV injection. This effect was significantly decreased when the PI3K-AKT-mTOR pathway was blocked. Wounds treated with ECV showed significantly higher thickness compared to control, along with better collagen deposition. These effects were reduced by LY294002 co-administration. It would have been desirable to investigate the effect of hAEC-ECV on vascularization and immunomodulation in this model. Interestingly, the route of administration in these studies was through injection in the surrounding tissue, rather than topical application.

#### Lung

The effect of hAEC-secretome on different lung pathologies was the topic of two extensive studies that used a bleomycin model of lung fibrosis or an allergic airway disease model. In the bleomycin model of lung fibrosis (BLM) (16), hAEC-ECV was administered on day 1 (early) and day 7 (late) after the bleomycin challenge. Administration on day 1 resulted in a significant reduction of CD4+ T cells and neutrophils in the spleen, and a percentage of CD4+ T cells and interstitial macrophages in the lung. Early administration also increased the expression of canonical Wnt targets including β-catenin, BMP4, BMPR1a, and NFATC1 compared to bleomycin injured mice. BASCs showed a significant reduction of c-MYC transcription, while significant upregulation of Axin 1 and Axin 2 was found in mucosal endothelial cells (MECs) in comparison to bleomycin controls, pointing to an inhibitory effect for Wnt/b-catenin signaling in MECs. In addition, FGFR1 was significantly elevated following ECV administration in comparison to bleomycin controls. No significant differences in CNND1, FOXM1, LEF1, PGK1, PTN, Sca1, TGFb, and WLS transcription were reported.

The effect of hAEC-ECVs administration on ongoing lung injury was evaluated by administering ECVs 7 days following bleomycin challenge (late intervention). Assessment at day 14 showed that the percentage of tissue-to-airspace ratio was significantly improved, and lung collagen content and αSMA expression were significantly reduced by ECV treatment. The number of BASCs per terminal bronchiole was significantly higher with hAEC-ECVs than bleomycin controls. hAEC-ECVs increased the percentage of ATII, but not ATI cells, fewer genes in MECs and BASCs were influenced by late ECV treatment in comparison to early treatment. No significant changes in gene expression were found in BASCs compared to control, while FZD6 expression was significantly increased in MECs (16).

Further investigating the effect of hAEC-ECVs in aged mice with bleomycin-induced fibrosis showed no evidence of bronchoalveolar hyperplasia, 2-fold reduction in pulmonary collagen, and a significant reduction in α-SMA^+^ cells in ECV-treated mice compared to bleomycin controls. The number of BASCs per terminal bronchiole, percentage of alveolar type I and II cells also showed a significant increase in the ECV-treated group compared to the control. Although the graphs show remarkable improvement in comparison to the hLF-exosome treated group, statistical comparison with this group was not provided by the authors (16).

These beneficial effects of hAEC-ECVs in the bleomycin-induced lung fibrosis mouse model were augmented by the second study (52), which showed a significant reduction in interstitial inflammation with ECV-treatment intranasally for 7 days. This time, the hAEC-ECV effect was also tested in an allergic airway disease (AAD) mouse model. Similarly, hAEC-ECVs significantly decreased airway inflammation in the AAD model. Best results in both models were achieved by combining hAEC-ECVs with serelaxin (S.C. through an osmotic pump), although it was not statistically significant compared to hAEC-ECVs alone. Interestingly, administration of hAEC-ECVs with serelaxin was significantly superior to hAECs with serelaxin in improving inflammation and reducing neutrophil and macrophage infiltration. ECV treatment alone (5 or 25 μg) normalized the increased airway epithelial thickening in the AAD model and the increased interstitial lung fibrosis in the BLM model. However, it only partially corrected the subepithelial ECM thickness in the AAD and BLM models, and total lung collagen concentration in the AAD model, which were normalized in the serelaxin + 25 μg exosome and serelaxin + hAEC groups.

Extracellular vesicles (ECVs) alone (at 5 or 25 μg) partly reduced airway thymic stromal lymphopoietin (TSLP)-associated epithelial damage, epithelial TGF-β1 expression, and subepithelial myofibroblast accumulation in the AAD model and induced myofibroblast accumulation in the BLM model. Twenty-five microgram of ECVs, but not 5-μg of ECVs, also partly reduced BLM-induced epithelial damage. Neither of the doses affected induced goblet cell metaplasia in the AAD model or BLM-induced interstitial TGF-β1 expression level. Serelaxin augmented the ability to reduce chronic AAD-induced goblet cell metaplasia, epithelial TGF-β1 expression, and subepithelial myofibroblast differentiation and was able to reduce BLM-induced lung epithelial damage and interstitial TGF-β1 expression. Combining serelaxin and 25-μg ECV also normalized chronic AAD and BLM-induced airway or lung epithelial damage. Combining serelaxin with 25-μg ECVs had a stronger influence in reducing the AAD or BLM-induced lung remodeling parameters measured compared to serelaxin + 1 ×10^6^ hAEC (52).

Interestingly, assessment of allergic hyperresponsiveness (AHR) in the AAD model and dynamic lung compliance in the BLM model by plethysmography showed that 25-μg ECVs alone, serelaxin alone, or hAEC + serelaxin could partially and significantly reduce the increased AHR in the AAD model, while alone had no effect. Combining serelaxin improved 5-μg ECVs ability to reduce AHR and resulted in normalization of AHR when combined with 25-μg ECVs. On the other hand, the significant decrease in lung compliance in the BLM model was corrected by all treatment groups in the study (52).

In summary, the various *in vivo* models focused mainly on the anti-fibrotic and immunomodulatory aspects of hAEC-secretome, especially the effect on macrophage polarization. For example, the anti-tumor effect of hAEC-secretome was not examined in an *in vivo* model. Furthermore, only few studies were able to point to a probable mediator for the ECV/CM effect. More *in vitro* studies are required to identify the effector miRNA or peptides in the case of ECVs.

## Challenges and Prospects

Cumulative evidence from published work shows that many of the desired effects of cells used in regenerative medicine are retained in their secretome including derived ECVs, i.e., of paracrine nature. One of the most studied cells in regenerative medicine generally and in ECV research specifically is the mesenchymal stem cells (MSCs). A similar approach to demonstrate the utility of hAEC-secretome was followed by several research groups as shown in this review. Direct comparison between hAECs and hAEC paracrine components has frequently demonstrated equal, or superior, the effect of the latter (6, 15, 19, 50, 51). The justification for this increasing body of research and interest in ECVs as a potential therapeutic tool is its advantages over cell-based therapy approaches (71). These advantages include: (1) avoiding troublesome aspects of cell therapy related to necrosis, cell fate, and vascular occlusion, (2) suitability for large-scale production and on-shelf formulation, and (3) direct uptake by surrounding cellular targets without engraftment, immunological, and vascularity related issues. Based on this and their natural biocompatibility, ECVs became the focus of many clinical trials and preclinical studies, mainly those derived from MSCs and dendritic cells, and an attractive business revenue for several companies (71).

However, several hurdles should be overcome before the ECV approach can move to the clinic. As a bioactive compound carrier, ECVs contain a dynamic cargo that is influenced not only by the cell of origin but also the culture condition and, moreover, the purification method (71, 72). This issue is further complicated when the cell of origin itself displays heterogeneity based on tissue of origin and isolation technique. The ECV proteomic profile was reported to be influenced by culture conditions (18) and the isolation method (13). In a recent study (18), the different formulations of the hAEC culture medium were shown to influence the total protein content, levels of tetraspanins, and several surface epitope markers in hAEC-ECVs. In the reviewed studies, the most evident variation in hAEC culture conditions was related to the serum content, which ranged from serum-free preparations to 10% serum supplementation. The percentage of serum supplementation, among other supplements, was shown to influence the total protein content (5 and 15% resulted in a 1.8 and 2-fold increase, respectively) and surface marker abundance including CD63, CD81, CD326, and CD41b (18). In a trial to standardize research methodologies related to ECVs, minimal requirements for ECV research have been proposed by the International Society for Extracellular Vesicles (ISEV) (11). These criteria are followed in most of the studies in the present review. The target of these criteria is to ensure that the utilized preparation qualifies for the ECV terminology and provides basic data regarding their source and preparation. The multiple variables in the process, including the condition of the placenta, the hAEC isolation protocol, the hAEC culture conditions, the ECV isolation technique, and the ECV characterization protocols make the standardization quite challenging. It was also shown that hAECs isolated from preterm or term placentae have a different expression of angiogenic factors and different paracrine functional outcomes (60). Hypoxia (27), oxidative stress (34, 63), and the presence of inflammatory mediators (60) were also reported to influence the expression profile and therefore can influence the resulting CM. Thus, for large-scale production, a minimal quality and characterization checklist would be required for both hAECs and their derivatives. However, the link between different components or markers of CM and ECVs on one side and the biological outcome on the other side is still being developed.

Regarding experimental research methodology, it should be noted that a proper control should be selected to appropriately assess the functional advantage of hAEC-ECVs or CM. For CM, an identical medium that did not come in contact with cells should be used for comparison rather than PBS or saline. Indeed, in one study (5), CM and control medium significantly decreased the number of LPCs in the liver. In the case of ECVs, if the target is to show the advantage of a particular cell of origin, a comparison with ECVs from another cell type is necessary depending on the type of advantage to be clarified. Human fibroblast-derived ECVs are commonly used for this target (16, 39). It is however desired to reach a consensus regarding a well-characterized ECV comparative control. In addition, the MISEV, in their 2018 update, recommended the use of the CM before and after the isolation of the ECVs as a control for ECVs in functional studies to exclude the effect of contaminants (11).

To allow for clinical use, the production of either ECVs or CM should conform with good manufacturing practices (GMPs). To achieve this goal, all the production materials and methods, including the type of cells, cultivation system, culture environment, culture medium, and dissociation enzyme, should be suitable for clinical application. In addition, proper purification and characterization that includes both the physical structure and bioactivity are necessary. The aspects related to GMP-grade ECV preparation have been reviewed elsewhere (72).

A technical challenge for ECV and CM administration is the delivery method (73). In experimental studies, these preparations are either injected directly into the target tissue or administered through the intraperitoneal and intravenous routes. The size of the experimental animals might be the reason for achieving beneficial effects after systemic administration because it allows for increasing the ECV dose to compensate for the off-target uptake. Studies have shown that systemically injected ECVs are rapidly taken by the monocyte phagocytic system and subsequently accumulate in the liver, spleen, lung, and bone marrow (74, 75). Strategies to inhibit the phagocytosis of systemically administered ECVs are being investigated (76, 77). Alternative target-specific delivery routes have also been investigated (78). In addition, repeated administration of ECVs is performed in many of the *in vivo* studies to achieve a similar effect as compared to cell transplantation. Therefore, other than the scenarios where the topical application could be feasible, e.g., corneal therapy and wound management, a mechanism for controlled release of ECVs into target tissue would be desired for clinical application. The uses of osmotic pumps (79) and hydrogel encapsulation were described for this target. Encapsulation with hydrogel has been proposed as a slow-release delivery system for ECVs (73, 80). The materials commonly used for ECV encapsulation range from natural polymers (e.g., collagen, gelatin, chitosan, hyaluronic acid, or alginate) to synthetic polymers [e.g., poly(ethylene glycol) (PEG), poly(lactic-co-glycolic acid) (PLGA), or poly(hydroxyethyl methacrylate) (pHEMA)] or their combination (80, 81). Methods to deliver these hydrogels to the pathological tissue would still need to be established. For example, intra-pericardial injection of ECV-hyaluronic acid hydrogel as a localized ECV release patch was investigated in a myocardial infarction model (82).

**Figure 4:**
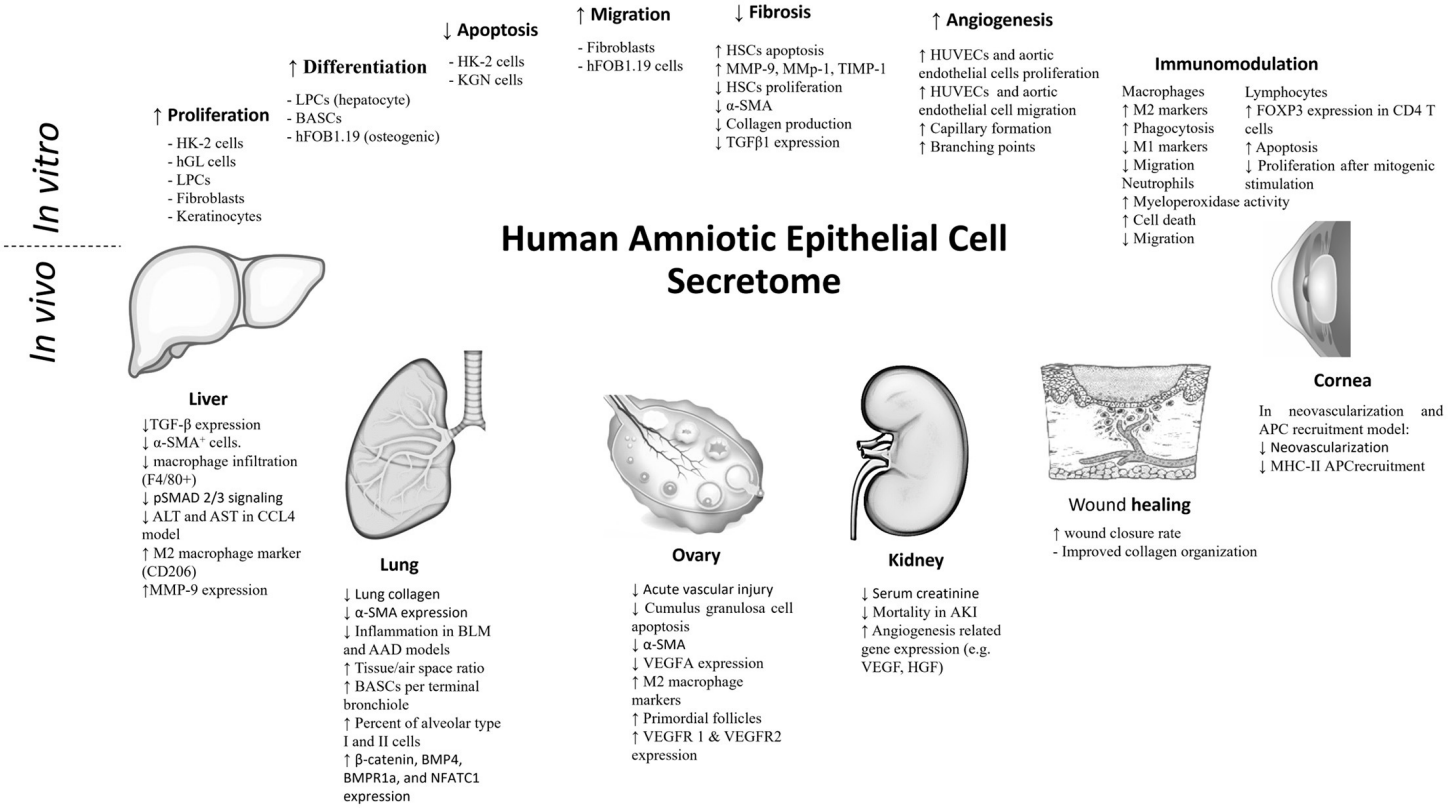
Paracrine effects of human amniotic epithelial cells.

In summary, published literature demonstrated that hAEC-secretome can, at least, replicate many of the hAEC effects *in vitro* and *in vivo* in different experimental animal models including liver fibrosis, lung fibrosis, wound healing, premature ovarian failure, corneal injury, and acute kidney injury. The effects of hAEC-ECVs and CM on target cells mainly fell into the angiogenic, immunomodulatory, anti-fibrotic, modulation of apoptosis, and improved cell migration categories (Figure 4). Comprehensive profiling of the hAEC-secretome content was included in few studies. However, dissection into the functional peptide or miRNA molecules and the associated molecular mechanisms is clearly desired.

## Author Contributions

IF: substantially contributed to the conception and design of the article, interpreting the relevant literature, and drafted the article. TM: revised it critically for important intellectual content. Both authors contributed to the article and approved the submitted version.

## Funding

This study was supported by the research grant from the Chairperson and the President of Nihon University and the Collaborative Research Grant from StemCell Institute Inc.

## Conflict of Interest

TM owns stock in Noveome Biotherapeutics, Inc. The remaining author declares that the research was conducted in the absence of any commercial or financial relationships that could be construed as a potential conflict of interest.

## Publisher's Note

All claims expressed in this article are solely those of the authors and do not necessarily represent those of their affiliated organizations, or those of the publisher, the editors and the reviewers. Any product that may be evaluated in this article, or claim that may be made by its manufacturer, is not guaranteed or endorsed by the publisher.

## Abbreviations

AAD: allergic airway disease
AFP: alpha-feto-protein
AHR: allergic hyper responsiveness
AMH: anti-Mullerian hormone
AMPK: AMP-activated protein kinase
AOD: autoimmune ovarian disease
ALT: alanine aminotransferase
α-SMA: α-smooth muscle actin
AST: aspartate aminotransferase
BASCs: bronchoalveolar stem cells
BDNF: brain-derived neurotrophic factor
BLM: bleomycin-induced lung fibrosis
BMP: Bone morphogenetic protein
CC10: club cell marker-10
CCL4: carbon tetrachloride
cffTF: cell-free fetal telomere fragments
CM: conditioned medium
CNTF: ciliary neurotrophic factor
DAT: dopamine transporter
ECV: extracellular vesicles
EVDM: extracellular vesicle depleted conditioned medium
GM-CSF: granulocyte-macrophage colony-stimulating factor
GMP: good manufacturing practice
GO: gene ontology
hAEC: human amniotic epithelial cells
hAMSC: human amniotic mesenchymal stem cells
HAS2: hyaluronic acid synthase 2
hDF: human dermal fibroblasts
hEF: adult foreskin fibroblast
hFB: human fibroblasts
hFOB: human fetal osteoblast
hGL: human granulosa-lutein
HLA: human leukocyte antigen
hLF: human lung fibroblasts
HMGB1: high mobility group box 1
HSC: hepatic stellate cells
hUVEC: human umbilical vein endothelial cells
IL: Interleukin
IL-1ra: interleukin-1 receptor antagonist
IPA: Ingenuity Pathway Analysis
ISEV: International Society for Extracellular Vesicles
IUA: intrauterine adhesion model
KEGG: Kyoto Encyclopedia of Genes and Genomes
LPC: liver progenitor cells
MECs: mucosal endothelial cells
MIF: macrophage inflammatory factor
MISEV: minimal information for studies of extracellular vesicles
MSC: mesenchymal stem cells
MVH: mouse vasa homolog
NASH: non-alcoholic steatohepatitis
NrCAM: neuronal cell adhesion molecule
OSM: oncostatin M
PGE2: prostaglandin E2
POF: premature ovarian failure
PPARγ: proliferator-activated receptor gamma
PROse: proteinase K
pZP3: zona pellucida protein 3 peptides
ROS: reactive oxygen species
TH: tyrosine hydroxylase
TRAIL: tumor necrosis factor related apoptosis inducing ligand.
